# A Systematic Review and Meta-Analysis of Efficacy, Cost-Effectiveness, and Safety of Selected Complementary and Alternative Medicine for Neck and Low-Back Pain

**DOI:** 10.1155/2012/953139

**Published:** 2011-11-24

**Authors:** Andrea D. Furlan, Fatemeh Yazdi, Alexander Tsertsvadze, Anita Gross, Maurits Van Tulder, Lina Santaguida, Joel Gagnier, Carlo Ammendolia, Trish Dryden, Steve Doucette, Becky Skidmore, Raymond Daniel, Thomas Ostermann, Sophia Tsouros

**Affiliations:** Clinical Epidemiology Methods Centre, Ottawa Hospital Research Institute, University of Ottawa Evidence-Based Practice Center, Box 208, Ottawa, ON, Canada K1H 8L6

## Abstract

*Background*. Back pain is a common problem and a major cause of disability and health care utilization. *Purpose*. To evaluate the efficacy, harms, and costs of the most common CAM treatments (acupuncture, massage, spinal manipulation, and mobilization) for neck/low-back pain. *Data Sources*. Records without language restriction from various databases up to February 2010. *Data Extraction*. The efficacy outcomes of interest were pain intensity and disability. *Data Synthesis*. Reports of 147 randomized trials and 5 nonrandomized studies were included. CAM treatments were more effective in reducing pain and disability compared to no treatment, physical therapy (exercise and/or electrotherapy) or usual care immediately or at short-term follow-up. Trials that applied sham-acupuncture tended towards statistically nonsignificant results. In several studies, acupuncture caused bleeding on the site of application, and manipulation and massage caused pain episodes of mild and transient nature. *Conclusions*. CAM treatments were significantly more efficacious than no treatment, placebo, physical therapy, or usual care in reducing pain immediately or at short-term after treatment. CAM therapies did not significantly reduce disability compared to sham. None of the CAM treatments was shown systematically as superior to one another. More efforts are needed to improve the conduct and reporting of studies of CAM treatments.

## 1. Introduction

Back pain is a general term that includes neck, thoracic, and lower-back spinal pain. In the majority of cases, the aetiology of back pain is unknown and therefore is considered as “nonspecific back pain”. Back pain is considered “specific” if its aetiology is known (e.g., radiculopathy, discogenic disease). Although back pain is usually self-limited and resolves within a few weeks, approximately 10% of the subjects develop chronic pain, which imposes large burden to the health-care system, absence from work, and lost productivity [1]. In a recent study, the direct costs of back pain related to physician services, medical devices, medications, hospital services, and diagnostic tests were estimated to be US$ 91 billion or US$ 46 per capita [2]. Indirect costs related to employment and household activities were estimated to be between US$ 7 billion and US$ 20 billion, or between US$25 and US$ 71 per capita, respectively [3–5]. One study published in 2007 showed that the 3-month prevalence of back and/or neck pain in USA was 31% (low-back pain: 34 million, neck pain: nine million, both back and neck pain: 19 million) [6]. 

The prevalence of back pain and the number of patients seeking care with complementary and alternative medicine (CAM) therapies in the US has increased over the last two decades [7]. The most prevalent CAM therapies for back and neck pain in the US are spinal manipulation, acupuncture, and massage [7]. The exact mechanisms of action of CAM therapies remain unclear. Recently, many randomized controlled trials (RCTs) have been conducted to study the effects of CAM therapies for back pain. The results of many systematic reviews [8–12], meta-analyses [13], and clinical practice guidelines [14–17] regarding the effectiveness of CAM therapies for back pain relative to no treatment, placebo, or other active treatment(s) in reducing pain and disability have been inconsistent. 

 The agency for healthcare research and quality (AHRQ) and the national center for complementary and alternative medicine (NCCAM) commissioned the University of Ottawa Evidence-based Practice Center (UO-EPC) to review and evaluate evidence regarding the effectiveness, cost-effectiveness, and safety of the most prevalent CAM therapies (i.e., acupuncture, manipulation, mobilization, and massage) used in the management of back pain. This technical report can be viewed at the AHRQ website (http://www.ahrq.gov/) [18]. The present paper summarizes the evidence from this technical report with a focus on a subset of studies reporting pain, disability, and harms outcomes compared between CAM therapies and other treatment approaches deemed relevant to primary care physicians (i.e., waiting list, placebo, other CAM therapies, pain medication, and physical therapy including exercise, electrotherapy and/or other modalities). The specific aims of this study were to systematically review and compare the efficacy, cost-effectiveness, and safety of acupuncture, manipulation, mobilization, and massage in adults (18 years or older) with neck or low-back pain.

## 2. Methods

### 2.1. Data Sources and Searches

We searched MEDLINE (1966 to February 2010), EMBASE (1980 to week 4 2010), the Cochrane Library (2010 Issue 1), CINAHL (1982 to September 2008), AMED (Allied and Complementary Medicine Database: 1985 to January 2010), Mantis (1880 to October 2008), and EBM Reviews—ACP Journal Club (1991 to August 2008). Two specialized CAM databases, the Index to Chiropractic Literature (ILC; October 2008) and Acubriefs (2008 October) were also searched. We searched using controlled vocabulary and keywords for conditions pertaining to neck pain, back pain, spinal diseases, sciatica, and various CAM interventions including acupuncture, electroacupuncture, needling, acupressure, moxibustion, manipulative medicine, manipulation, chiropractic, and massage. (Appendix A: Complete search strategies for each database). The searches were not restricted by language or date. We also reviewed reference lists of eligible publications.

### 2.2. Study Selection

RCTs reporting efficacy and/or economic data of CAM therapies in comparison with no treatment, placebo, or other active treatments in adults with low-back, neck, or thoracic pain were eligible. Nonrandomized controlled trials and observational studies (e.g., cohort, case-control, cross-sectional) reporting harms were also included. Reports published in English, German, Dutch, Chinese, Japanese, Italian, French, Portuguese, and Spanish were eligible for inclusion. Systematic and narrative reviews, case reports, editorials, commentaries or letters to the editor were excluded. 

 Two independent reviewers screened the titles and abstracts and later reviewed the full-text reports of potentially eligible records. Discrepancies were resolved by consensus.

### 2.3. Data Extraction and Risk of Bias Assessment

Two independent reviewers extracted data on study and population characteristics, treatment, study outcomes, and duration of posttreatment followup. The abstracted data were verified and conflicts were resolved by consensus. 

 Treatment efficacy outcomes were pain intensity (e.g., Visual Analog Scale-VAS, McGill Pain Questionnaire-MPQ) and disability (e.g., Roland-Morris Disability Questionnaire-RMDQ, Northwick Park Neck Pain Questionnaire-NPQ, Pain Disability Index-PDI, Oswestry Disability Index). The timing of posttreatment followup for outcomes was ascertained and categorized into four groups: immediate, short- (<3 months), intermediate- (3 to 12 months), and long-term (>12 months) posttreatment followup. Harms (e.g., any adverse event, withdrawals due to adverse events, specific adverse events) were extracted as proportions of patients with an event. 

 For cost-effectiveness analysis, data was extracted on: (a) costs to the health care sector, (b) costs of production loss, (c) costs in other sectors, (d) patient and family costs, and (e) total costs. 

 The risk of bias for RCTs was assessed using the 13-item criteria list (item rating: Yes, No, Unclear) recommended in the Updated Method Guidelines for Systematic Reviews in the Cochrane Collaboration Back Review Group [19]. The risk of bias for each RCT was classified into three groups: good (score: 4), fair (score: 2-3), and poor (score: 0-1) depending on the number of “Yes” ratings (score range: 0–4) across the four domains (treatment allocation concealment, balance in baseline characteristics, blinding, and number/reasons for dropouts). Assessment of quality of reporting in observational studies was done by using the modified 27-item tool of Downs and Black [20]. Methodological quality of economic studies was determined using the 19-item Consensus Health Economic Criteria [21].

### 2.4. Rating the Strength of the Body of Evidence

The overall strength (i.e., quality) of evidence was assessed using the grading system outlined in the Methods guide prepared for the AHRQ Evidence-based Practice Center (EPC) program [22]. The grading was based on four domains: overall risk of bias, consistency, directness, and precision (applied to pooled results only). The overall risk of bias (high, medium, and low) was derived by averaging the risk of bias (good, fair, and poor) across individual trials. If evidence consisted of only one study (or multiple studies of the same risk of bias score), then the risk of bias for individual study corresponded to the overall risk of bias for this evidence as follows: “poor” (score: 0 or 1) = risk of bias (high), “fair” (score: 2 or 3) = risk of bias (medium), and “good” (score: 4) = risk of bias (low). In case of evidence consisting of multiple studies with different risk of bias scores (studies that scored “poor”, “fair”, and “good” mixed together), the mean risk of bias score (i.e., mean number of “Yes”) was calculated and the overall risk of bias was defined as “high” (mean score < 2), “medium” (2 ≤ mean score < 4), and “low” (mean score = 4). Consistency was judged based on qualitative assessment of forest plots of meta-analyses (direction and 95% confidence intervals of the effects in individual trials). Results were considered consistent when statistically significant or nonsignificant effects in the same direction were observed across trials. When pooling was not possible, consistency was judged based on qualitative summary of the trial results. The pooled estimate with relatively narrow 95% CIs leading to clinically uniform conclusions was considered as “precise evidence”. Relevant health outcomes (pain, disability) were defined as “direct” as opposed to intermediate or surrogate outcomes (“indirect”). The grade of the evidence for a given outcome was classified into four groups: high, moderate, low, or insufficient (no evidence). The initial “high” grade was reduced by one level (from high to moderate) for each of the domains not met (i.e., overall risk of bias, consistency, directness, precision) and by two levels in case of high risk of bias (e.g., from high to low grade).

### 2.5. Data Synthesis and Analysis

The results were grouped according to the type of experimental intervention (e.g., acupuncture, manipulation, mobilization, massage), pain location in spinal region (low-back, neck, head, thorax), duration of pain (acute/subacute, chronic, mixed, unknown), and cause of pain (specific, nonspecific). Study, treatment, population, and outcome characteristics were summarized in text and summary tables. 

We meta-analyzed RCTs with similar populations (demographics, cause, location, and duration of spinal pain), same types of experimental and controls treatments, and outcomes measured with the same instruments (and scale) at similar posttreatment followup time points. The meta-analyses of pain were based on the Visual Analogue Scale (VAS; score range: 1–10). The random-effects models of DerSimonian and Laird were used to generate pooled estimates of weighted end point mean difference (WMDs) with 95 percent confidence intervals (95% CIs). Statistical heterogeneity was evaluated using the Chi-square test and the *I* ^2^ statistic (low: 25.0%; moderate: 50.0%; high: 75.0%). Subgroup (e.g., patients' age, gender) and sensitivity (e.g., trial quality) analyses were planned to investigate the sources of heterogeneity. 

The degree of clinical importance for the observed differences in pain scores between the treatment groups was specified according to the Updated Method Guidelines of Cochrane Collaboration Back Review Group [19]: small (WMD < 10% of the VAS scale), medium (10% ≤ WMD < 20% of the VAS scale), and large (WMD ≥ 20% of the VAS scale). 

Publication bias was examined through visual inspection of funnel plot asymmetry and the Egger's regression-based method [23].

### 2.6. Role of the Funding Source

This topic was nominated by NCCAM and selected by AHRQ. A representative from AHRQ served as a Task Order Officer and provided technical assistance during the conduct of the full evidence report and comments on draft versions of the full evidence report. AHRQ did not directly participate in the literature search, determination of study eligibility criteria, data analysis or interpretation, preparation, review, or approval of the paper for publication.

## 3. Results

**Figure 1:**
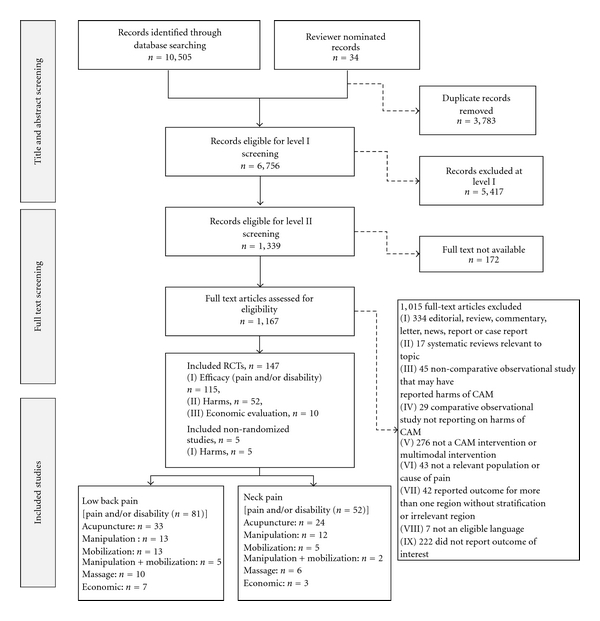
Flow diagram.

Our literature search identified 152 unique studies: 147 RCTs and 5 nonrandomized studies (1 controlled trial and 4 observational) were included in the review (Figure 1). One hundred and fifteen RCTs reported data on efficacy (pain and disability) and/or harms. Additionally, 23 RCTs that did not report pain and disability outcomes provided data on harms. Five nonrandomized studies reported harms. Ten RCTs reported on cost-effectiveness (one of the 10 RCTs also reported efficacy). 

### 3.1. Study Characteristics

The included studies were published between 1978 and 2009. The studies were published in English (74.5%), Chinese (3.3%; all acupuncture) [24–28], German (<1.0%; massage of lumbar region) [29], Japanese (2.6%; all acupuncture) [30–33], and one in Spanish (spinal mobilization) [34]. All 10 reports of economic evaluation of CAM treatments were published in English [35–44].

### 3.2. Population Characteristics

The majority of trials (>90%) included adult men and women aged 18–65 years. Six trials included adults aged 55 years or older [45–50]. In total, 61% of all studies included subjects with nonspecific pain. About 85%, 14%, and 12% of acupuncture, spinal manipulation/mobilization, and massage trials, respectively, enrolled subjects with nonspecific cause of back pain. The remaining trials enrolled subjects with specific causes of back pain (e.g., disc perturbation, whiplash, myofascial pain, cervicogenic headache, or underlying neurological causes).

### 3.3. Treatment Characteristics

#### 3.3.1. Acupuncture Studies

A large variety of methods of acupuncture treatments were used to compare the effect of acupuncture and control treatments. The control treatments in these trials included active (i.e., physical modalities and exercise) or inactive treatments (i.e., placebo, no treatment). The treatment providers were trained or licensed acupuncturists, general practitioners or physicians with especial training in acupuncture, neuropathy physicians, general practitioners, and trained physiotherapists. In the majority of Chinese trials, the treatment provider was referred as “therapist”.

#### 3.3.2. Manual Treatment Studies

Interventions were provided by experienced and licensed chiropractors, physical therapists, general practitioners, licensed or qualified manual therapy practitioners, nonspecified clinicians, neurologists or rheumatologists, folk healers, and osteopaths.

#### 3.3.3. Massage Studies

Treatment providers were licensed or experienced massage therapists, physical therapists, reflexologists, acupressure therapists, folk healers, general practitioners, manual therapists, experienced bone setters, and chiropractic students.

### 3.4. Risk of Bias Assessment

#### 3.4.1. RCTs Reporting Efficacy and Harms

**Figure 2:**
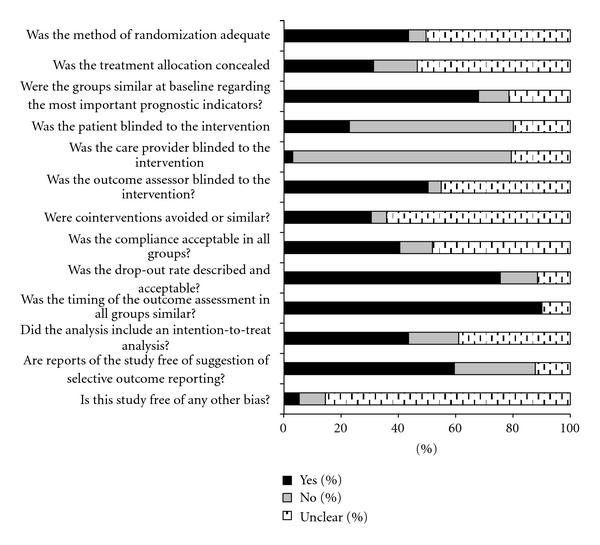


The risk of bias was assessed for 131 RCTs. Overall, the methodological quality of the RCTs was poor (median score = 6/13; inter-quartile range: 4, 7). Only 71 (54%) of the studies scored 6 or higher based on the 13 items of risk of bias tool. An adequate method of randomization was described in 57 (43.5%) studies. The remaining 74 studies either did not report the method used for randomization (*n* = 8; 6.0%) or the method used was not clearly described (*n* = 66; 50.0%). Concealment of treatment allocation was judged as adequate for 41 (31.3%) of RCTs and inadequate for 20 (15.3%) of RCTs (Table 1 and Figure 2).

#### 3.4.2. RCTs Reporting Economic Evaluation

Of the 10 studies reporting cost-effectiveness data, 3 studies collected costs appropriate to their chosen perspective. Two studies did not state the perspective adopted for the economic evaluation. Most studies measured costs using diaries, questionnaires, practice/insurance records, and valued costs appropriately using published sources. Most studies conducted an incremental cost-effectiveness analysis. The length of followup across the studies was at least one year. In one study with a length of followup of more than one year, discounting was undertaken [39].

#### 3.4.3. Observational Studies (Cohort and Case-Control)

The objectives and the main outcome (an adverse event) of the 4 studies were well described. The studies had a large sample size ranging from 68 to 3982 subjects, providing sufficient power to detect clinically important effects.

### 3.5. Efficacy of Acupuncture for Low-Back Pain

This section included 33 trials (Table 2 for efficacy results and evidence grading (Appendix B)) [26, 30–33, 35, 36, 41, 45, 47–49, 51–73]. One study [26] was published in Chinese and four studies were published in Japanese [30–33]. The trials were conducted in China (37%), Europe (United Kingdom, Germany, Ireland, and Sweden; 35%), and USA (28%). 

#### 3.5.1. Acupuncture versus Inactive Treatment

**Figure 3:**
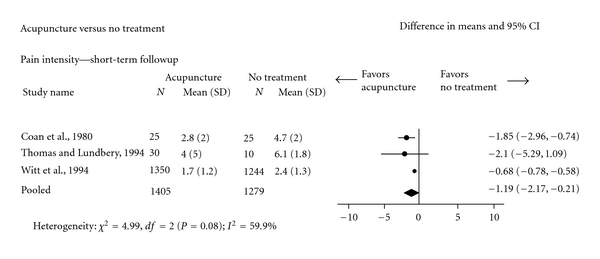
Acupuncture versus no treatment for chronic nonspecific low-back pain (Pain intensity: Visual Analogue Scale).

One meta-analysis (Figure 3) showed that subjects with chronic nonspecific LBP receiving acupuncture had statistically significantly better short-term posttreatment pain intensity (3 trials; pooled VAS: −1.19, 95% CI: −2.17, −0.21) [48, 52, 74] and less immediate-term functional disability (1 trial) [51] compared to subjects receiving no treatment. 

**Figure 4:**
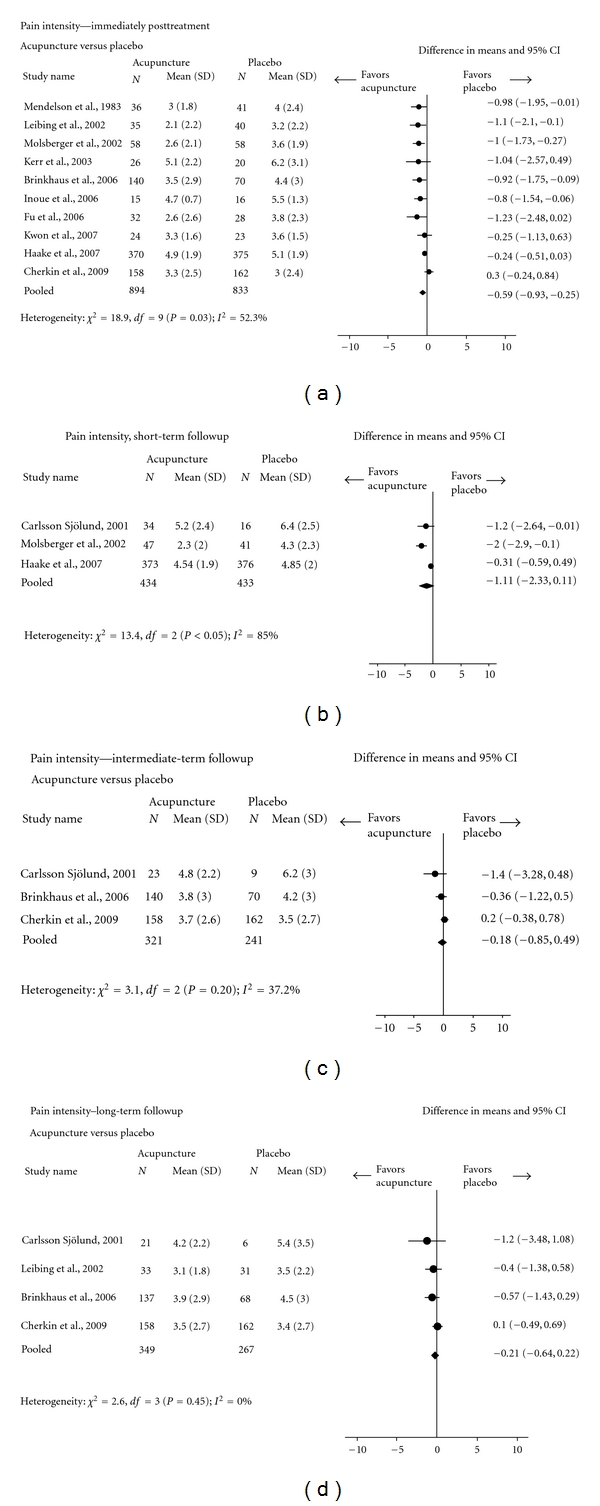
Acupuncture versus placebo for chronic nonspecific low-back pain (Pain intensity: Visual Analogue Scale).

Trials comparing acupuncture to placebo yielded inconsistent results with respect to pain intensity. For subjects with acute/subacute nonspecific LBP, acupuncture did not significantly differ from placebo on pain or disability outcomes [31, 53]. In a meta-analysis (Figure 4) of subjects with chronic nonspecific LBP, acupuncture compared to placebo led to statistically significantly lower pain intensity, but only for the immediate-posttreatment followup (10 trials; pooled VAS: −0.59, 95% CI: −0.93, −0.25) [51, 55, 56, 58, 59, 61–65, 67]. The mean pain intensity scores in the acupuncture and placebo groups were not significantly different at short- [51, 55, 56, 58] intermediate-[51, 54, 58], and long-term [51, 54, 63, 67] followups. Acupuncture did not significantly differ from placebo in disability [62, 67]. Trials using sham-TENS, sham-laser, or placebo medication tended to produce results in favor of acupuncture in relation to pain intensity and disability compared to trials using sham-acupuncture.

#### 3.5.2. Acupuncture versus Active Treatment

Two meta-analyses showed that acupuncture did not significantly differ from pain medication in reducing immediate posttreatment pain (4 trials; VAS score) [49, 69–71] or disability (2 trials; Oswestry score) [69, 70] in patients with chronic nonspecific low-back pain (Data is not presented in Figures). 

**Figure 5:**
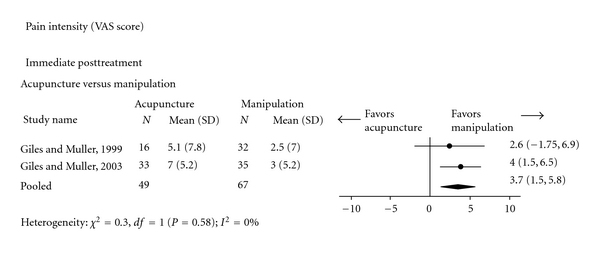
Acupuncture versus Manipulation for chronic nonspecific low-back pain (Pain intensity: Visual Analogue Scale).

 Another meta-analysis (Figure 5), based on subjects with chronic nonspecific low-back pain, indicated that manipulation was significantly better than acupuncture in reducing pain immediately after the treatment (2 trials; VAS score: 3.70, 95% CI: 1.50, 5.80) [69, 70]. 

 One trial showed that subjects receiving acupuncture had significantly better immediate posttreatment pain and disability than subjects receiving a combination of physical modalities (the light, electricity, heat) [26]. 

Massage was significantly better than acupuncture in reducing pain intensity and disability at immediate- or long-term followups for subjects with chronic nonspecific LBP [36]. 

 Subjects with chronic nonspecific LBP receiving acupuncture compared with those receiving usual care (analgesics, anti-inflammatory drugs, primary care, recommendation for physical therapy visits) had significantly better short-/intermediate-term posttreatment pain intensity (2 trials; VAS score) [47, 67] and disability (2 trials; RMDQ score) [47, 67]. However, in subjects with acute nonspecific LBP, posttreatment disability (RMDQ) was not significantly different between the acupuncture plus usual care (limited bed rest, education, and nonsteroidal anti-inflammatory drugs, activity alterations) and usual care alone groups (1 trial) [41]. 

### 3.6. Efficacy of Acupuncture for Neck Pain

This section included 24 trials (Table 3 for efficacy results and evidence grading (Appendix B)) [24, 27, 28, 69, 70, 72, 75–94]. About 38% of studies were conducted in Europe (Germany, Spain, Sweden, Turkey, United Kingdom), 17% in Australia, 8% in Japan, and 8% in the USA. The remaining 29% of trials were conducted in Brazil, South Korea, and Taiwan. All studies in this section were published in English language.

#### 3.6.1. Acupuncture versus Inactive Treatment

In one trial of subjects with unknown duration of myofascial neck pain [75], acupuncture was significantly better than no treatment in reducing pain intensity (McGill pain questionnaire) shortly after the end of treatment (mean change from baseline: −15.2 ± 13.3 versus −5.3 ± 8.7, *P* = 0.043). There was no evidence comparing acupuncture to no treatment in subjects with neck pain of acute/subacute, chronic, and mixed duration. 

**Figure 6:**
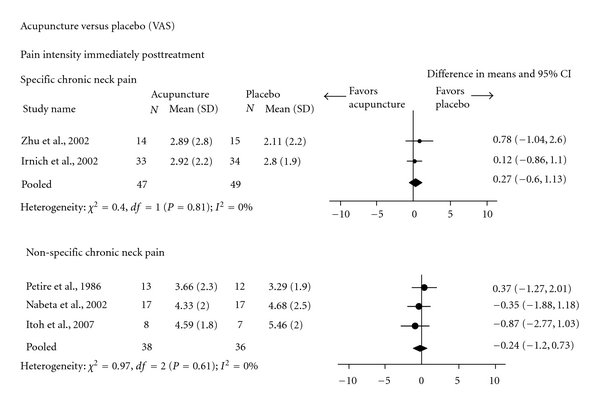
Acupuncture versus placebo for chronic-specific and nonspecific neck pain (Pain intensity: Visual Analogue Scale).

 Two meta-analyses (Figure 6) indicated no significant difference between acupuncture and sham-acupuncture in subjects with chronic-specific (two trials; VAS score: 0.27, 95% CI: −0.60, 1.13) [77, 78] or nonspecific pain (three trials; VAS score: − 0.24, 95% CI: −1.20, 0.73) [80–82] for immediate posttreatment pain intensity. Similarly, one trial of subjects with mixed specific pain showed no significant difference between acupuncture and placebo in reducing pain intensity (VAS score) or improving disability immediately after treatment [88]. There was no evidence comparing acupuncture to placebo in subjects with acute/subacute duration of neck pain.

#### 3.6.2. Acupuncture versus Active Treatment

There were inconsistent results for immediate- or short-term posttreatment pain intensity between acupuncture and pain medication in subjects with chronic and unknown duration of pain (8 trials) [28, 69, 70, 87, 89–92]. For subjects with chronic nonspecific pain, acupuncture was significantly better in reducing pain than NSAIDs immediately after treatment [91]. Similarly, in two trials, acupuncture was significantly more effective than injection of Lidocaine in short-term followup for treatment of unknown nonspecific neck pain [28, 92]. In other five trials, there was no significant difference between acupuncture and pain medication [69, 70, 87, 89, 90].

There were inconsistent results for immediate- or short-term posttreatment pain intensity between acupuncture and spinal manipulation for chronic pain (3 trials) [24, 70, 72]. Immediate/short-term posttreatment disability score (NDI) was better in manipulation than acupuncture groups of subjects with chronic nonspecific pain (2 trials) [69, 70]. 

Acupuncture did not differ from mobilization [93] or laser therapy [95, 96] in short-term posttreatment pain intensity or disability (3 trials). 

 In one trial [76], acupuncture was significantly better than massage in reducing pain intensity at short-term posttreatment followup (mean VAS score change from baseline: 24.22 versus 7.89, *P* = 0.005).

### 3.7. Efficacy of Manipulation for Low-Back Pain

This section included 13 studies using manipulation alone [69, 70, 72, 97–108]. (Table 4 for efficacy results and evidence grading (Appendix B)). About 62% of studies were conducted in North America (USA and Canada), 15% in Australia, and the remaining 23% in Europe (United Kingdom, Italy), and (Egypt).

#### 3.7.1. Manipulation versus Inactive Treatment

In subjects with acute/subacute [97, 99–101, 109] and mixed duration [98, 104] nonspecific LBP, manipulation was significantly more effective than placebo [97, 99–101, 104, 109] or no treatment [97, 98] in reducing pain intensity immediately or in the short-term following treatment. There was no significant difference between manipulation and placebo in posttreatment pain disability. In subjects with chronic nonspecific LBP, manipulation was significantly more effective than placebo in reducing pain intensity (VAS score) immediately or short-term after the end of treatment [100, 102, 103].

#### 3.7.2. Manipulation versus Active Treatment

Manipulation was significantly better (in immediate posttreatment pain) or no different (in intermediate-term posttreatment pain) than pain medication in improving pain intensity [69, 70]. Manipulation did not differ from pain medication in reducing pain intensity at short- and intermediate-term followup after treatment [100]. 

In older subjects with mixed LBP duration, spinal manipulation was significantly better than medical care (exercise, bed rest, analgesics) in improving immediate and short-term posttreatment disability (Oswestry), although no significant difference could be found in pain intensity [106].

In two large trials [110, 111], subjects receiving combination of manipulation and exercise or manipulation and best care by general practitioner (analgesics or muscle relaxants) improved in pain and disability compared to subjects with no spinal manipulation treatment.

### 3.8. Efficacy of Manipulation for Neck Pain

This section included 12 trials (Table 5 for efficacy results and evidence grading (Appendix B)) [69, 70, 72, 112–121]. About half of the studies were conducted in North America (USA and Canada), 16% in Europe (Germany, Spain) and the remaining 34% of the studies in Australia.

#### 3.8.1. Manipulation versus Inactive Treatment

There was no significant difference in reducing pain intensity between manipulation and “no treatment” groups in immediate-term posttreatment in subjects with unknown nonspecific pain (1 trial) [112].

Subjects with acute, subacute, chronic or unknown neck pain receiving manipulation had significantly better posttreatment pain (4 trials) [113–116] and disability (1 trial) [116] compared to those taking placebo.

#### 3.8.2. Manipulation versus Active Treatment

In two trials [69, 70], manipulation was significantly better than medication (e.g., NSAIDs, Celebrex, Vioxx, Paracetamol) in reducing pain intensity and improving disability score at immediate/short-term followup. 

 In subjects with acute/subacute nonspecific pain there was no statistically significant difference between manipulation and mobilization immediately after treatment (1 trial) [114]. In subjects with mixed duration nonspecific neck pain, manipulation was statistically significantly more effective than mobilization in reducing pain immediately after treatment (2 trials) [119, 120]. In one trail [121], there were no clinically or statistically significant differences between manipulation and mobilization in reducing pain or improving disability at intermediate term followup [121].

### 3.9. Efficacy of Mobilization for Low-Back Pain

This section included 13 trials (Table 6 for efficacy results and evidence grading (Appendix B)) [25, 34, 122–134]. About 30% of the trials were conducted in the US, 54% in Europe (Finland, United Kingdom, Sweden, Spain), and 16% in Australia, Thailand, and China. Two studies were published in either Spanish [34] or Chinese [25].

#### 3.9.1. Mobilization versus Inactive Treatment

Subjects with acute/subacute [122] and chronic nonspecific LBP [34] receiving mobilization experienced significantly improved pain intensity VAS, MPQ [122] compared to subjects not receiving any treatment, immediately posttreatment [34, 122]. Results regarding disability (RMDQ, Oswestry) were inconsistent, showing either a significant difference in favour of mobilization [34] or no difference [123] between mobilization and no treatment. In one trial of subjects with mixed duration of LBP, there was no significant difference in pain intensity immediately posttreatment compared to no treatment [124]. 

 In subjects with acute/subacute specific (pelvic joint dysfunction) [125, 126] and nonspecific mixed duration LBP [127] there were no significant differences in pain intensity (VAS) between mobilization and placebo groups immediately [125, 126] and in the short-term [127] after treatment.

#### 3.9.2. Mobilization versus Active Treatment

In two meta-analyses, subjects with chronic nonspecific LBP receiving mobilization (traditional bone setting) compared to physiotherapy (massage, stretching, trunk exercise) had significantly lower pain intensity (pooled VAS score: −0.50, 95% CI: −0.70, −0.30) [128–130] and disability (pooled Oswestry score: −4.93, 95% CI: −5.91, −3.96) [128–130] immediately posttreatment. 

In one trial, the manipulation group had a significantly better disability score compared to the mobilization group immediately posttreatment [132]. In two trials, mobilization was shown either significantly worse than [133] or no different [25] from massage in reducing short-term posttreatment pain intensity amongst subjects with chronic nonspecific [133] or unknown duration of LBP [25]. 

The immediate- posttreatment pain intensity (VAS) [134] and disability (Oswestry) [131] did not significantly differ between mobilization and exercise in trials with mixed duration of LBP (2 trials) [131, 134]. In a trial including subjects with nonspecific pain of mixed duration, mobilization was significantly superior to exercise in reducing disability (Oswestry) at intermediate- and long-term posttreatment followup [131].

### 3.10. Efficacy of Mobilization for Neck Pain

This section included 5 trials (Table 7 for efficacy results and evidence grading (Appendix B)) [114, 135–138]. The trials were conducted in Europe (Finland, Germany, the Netherlands) and Canada.

#### 3.10.1. Mobilization versus Inactive Treatment

In two trials [135, 136], subjects with chronic or mixed nonspecific pain receiving mobilization had significantly lower pain intensity compared to no treatment. Mobilization was significantly better than placebo in subjects with acute/subacute nonspecific pain (1 trial) [114], but did not differ from placebo in subjects with chronic nonspecific pain (1 trial) [135].

#### 3.10.2. Mobilization versus Active Treatment

Mobilization was significantly better than massage [137] or physiotherapy (massage, stretching and exercise) [137, 138] in improving pain (VAS score) and disability (NDI) in subjects with chronic and mixed nonspecific pain at intermediate-term posttreatment followup (2 trials) [137, 138]. Subjects with nonspecific pain of mixed duration in the mobilization and continued general practitioner care (analgesics, counselling, and education) groups had similar posttreatment pain intensity (VAS) and disability (NDI) [138].

### 3.11. Efficacy of Massage for Low-Back Pain

This section included 10 trials (Table 8 for efficacy results and evidence grading (Appendix B)) [29, 139–147]. About half of the studies were conducted in Europe (Belgium, Germany, United Kingdom), 30% in North America (USA and Canada), and 20% in Taiwan. One study was published in German Language [29].

#### 3.11.1. Massage versus Inactive Treatment

Subjects with acute/subacute nonspecific LBP receiving massage had significantly better pain intensity (VAS, MPQ) and disability (Oswestry) compared to no treatment (1 trial) [139] or placebo (2 trials) [139, 141] immediately or short-term after the end of treatment. In subjects with chronic nonspecific LBP, massage did not significantly differ from no treatment [140] or placebo [142] in improving immediate or intermediate-term posttreatment pain intensity (SF-36 pain scale, MPQ; 2 trials) [140, 142] or disability (Oswestry, RMDQ; 2 trials) [140, 142].

#### 3.11.2. Massage versus Active Treatment

In two meta-analyses, massage was significantly better in reducing pain compared to relaxation (2 trials, pooled VAS score: −1.27, 95% CI: −2.46, −0.08) [145, 146] or physical therapy (2 trials; pooled VAS score: −2.11, 95% CI: −3.15, −1.07) [143, 144] immediately after treatment of subjects with chronic nonspecific LBP. 

 In subjects with chronic nonspecific LBP, there was no significant difference between receiving massage and usual care (advice and exercise) in improving pain (VAS score) or disability (RMDQ) intermediate-term after the end of treatment (1 trial) [147].

### 3.12. Efficacy of Massage for Neck Pain

This section included 6 trails (Table 9 for efficacy results and evidence grading (Appendix B)) [76, 148–152]. Four trials were conducted in Europe (Finland, Germany, the Netherlands) and two trials in North America (USA and Canada). 

#### 3.12.1. Massage versus Inactive Treatment

Massage compared to no treatment significantly improved pain intensity (NPQ, VAS scores) in subjects with chronic or unknown duration of nonspecific pain, immediately after the end of treatment (2 trials) [148, 150]. Subjects with acute/subacute, chronic, or unknown duration of nonspecific pain receiving massage had significant improvement in pain intensity (≥2-point decrease on NRS-11, VAS) compared to subjects receiving placebo (2 trials), immediately after treatment [76, 151]. 

#### 3.12.2. Massage versus Active Treatment

In subjects with chronic nonspecific pain, massage compared to exercise significantly improved disability (NPQ) immediately after the treatment (1 trial) [148].

### 3.13. Efficacy of Combination of Manipulation and Mobilization for Low-Back Pain

This section included 5 trials (Table 10 for efficacy results and evidence grading (Appendix B)) [153–158]. The studies were conducted in Europe (the Netherlands, United Kingdom, and Norway), Australia, and the USA.

#### 3.13.1. Manipulation Plus Mobilization versus Inactive Treatment

Subjects with acute/subacute nonspecific LBP receiving manipulation plus mobilization were not significantly better than subjects who received a double placebo (sham manipulation and placebo analgesic) (1 trial) [159].

#### 3.13.2. Manipulation Plus Mobilization versus Active Treatment

Manipulation plus mobilization was significantly better in reducing pain than physiotherapy (exercise, massage, heat, electrotherapy, ultrasound) in subjects with mixed duration of LBP (1 trial) [154], better than hospital outpatient treatment in subjects with nonspecific LBP of unknown duration (1 trial) [157], and better than exercise for pain (VAS) and disability (RMDQ) in subjects with chronic nonspecific LBP (1 trial) [158]. However, there was no difference between manipulation plus mobilization and usual care (analgesics, muscle relaxants, instruction in proper back care, life-style recommendations, and exercise) in subjects with mixed duration of nonspecific LBP (1 trial) [156].

### 3.14. Efficacy of Combination of Manipulation and Mobilization for Neck Pain

This section included 2 studies (Table 11 for efficacy results and evidence grading (Appendix B)) [155, 160–162]. The studies were conducted in Australia and The Netherlands.

#### 3.14.1. Manipulation Plus Mobilization versus Inactive Treatment

In one trial, in subjects with chronic nonspecific pain, spinal manipulation plus mobilization was significantly better in reducing pain intensity and the frequency of headache than no treatment (*P* < 0.001) [160, 162].

#### 3.14.2. Manipulation Plus Mobilization versus Active Treatment

In one trial [162], spinal manipulation plus mobilization did not differ from exercise alone in reducing headache frequency (number per week), intensity (VAS score: 0–10) and neck pain (percentage of patients who improved ≥50% on a 10-point MPQ scale). However, the combination was significantly better than physiotherapy (exercise, massage, heat, electrotherapy, ultrasound, shortwave diathermy) in reducing pain intensity (1 trial) [155, 161].

### 3.15. Extent of Publication Bias

**Figure 7:**
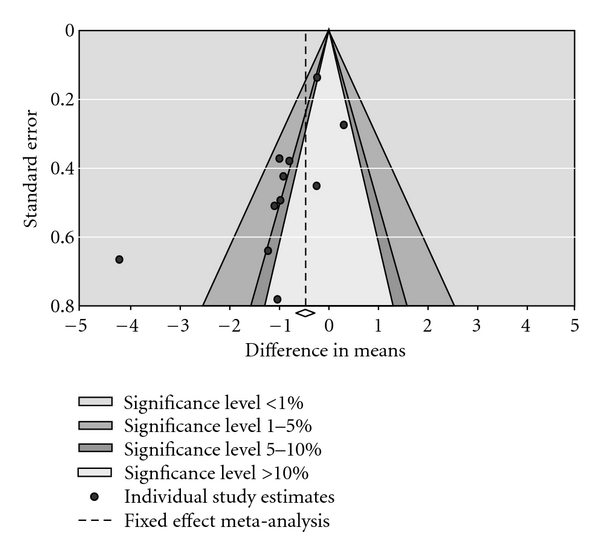
Funnel plot of trials comparing VAS score (acupuncture versus placebo).

Visual inspection of the funnel plot (Figure 7) for the acupuncture trials comparing immediate posttreatment mean VAS scores between acupuncture and placebo treatment groups suggested some degree of asymmetry. Specifically, there was a relative lack of trials with negative results (i.e., fewer trials in areas of statistical nonsignificance), indicating a potential for publication bias. The Egger's regression-based analysis [23] yielded a statistically significant result (*P* = 0.03).

### 3.16. Cost-Effectiveness

This section included results from 10 studies of full economic evaluations of acupuncture (low-back pain: 2 studies, neck pain: 1 study), spinal manipulation (low-back pain: 4 studies, neck pain: 2 studies), and massage (1 study) for low-back [35, 37–40, 43, 44] and neck pain [163–165].

#### 3.16.1. Acupuncture—Low-Back Pain

Two economic evaluations showed that acupuncture was cost-effective compared to usual care and compared to no treatment in patients with chronic low-back pain [35, 39]. However, in both studies health gains were small and one study used no treatment control group and had only 3 months of followup.

#### 3.16.2. Acupuncture—Neck Pain

One study [164] showed that in subjects with chronic neck pain acupuncture use was associated with significantly higher total costs compared to usual care ($1,565 versus $1,496).

#### 3.16.3. Manipulation—Low-Back Pain

There were no differences in costs between manual therapy, general practitioner care (rest, sick leave, direct prescription, advice about posture, and information about nature of the pain), and intensive therapy for acute LBP [44]. Costs were higher for manipulation compared with medical care (analgesics or muscle relaxants) without producing better clinical outcomes for patients with mixed duration of LBP [37]. This was associated with significantly more visits to chiropractic care than medical care. Spinal manipulation in addition to general practitioner care (active management; back book) was relatively cost-effective compared to general practitioner care alone for patients with subacute and chronic LBP [40]. In chronic LBP patients, there were no differences in costs between physician consultation, spinal manipulation plus stabilizing exercises, and physician consultation alone [43]. Results are difficult to compare due to differences in health care systems, perspectives, interventions, populations, and methods used.

#### 3.16.4. Manipulation—Neck Pain

One study [163] in subjects with neck pain found that pulsed short-wave diathermy was less cost-effective compared with manual therapy or exercise/advise. In another study [165], manual therapy was less costly and more effective than physiotherapy (functional, active and postural or relaxation exercises, and stretching) or general practitioner care (advice and exercise).

#### 3.16.5. Massage—Low-Back Pain

One study [38] reported an economic evaluation of therapeutic massage, exercise, Alexander technique, and usual general practitioner care (counselling, education, and pain medication) in patients with chronic low-back pain showing that massage was more costly and less effective than usual care by the general practitioner.

### 3.17. Harms of CAM Therapies

Reports of 57 trials provided data on harms. The reporting of harms was poor across the studies (e.g., lack of consistency, not detailed, not comparable). No definitions were presented. Therefore, rates of adverse events between the different interventions could not be meaningfully compared.

#### 3.17.1. Acupuncture—RCTs

The reported events in RCTs [35, 36, 41, 45, 47, 49–51, 55, 56, 61–63, 63, 67, 69, 73, 76, 78, 80, 81, 83, 84, 86, 89, 92, 166–179] were mostly of moderate and transient nature. Most commonly reported events were soreness/pain at the site of needling, bruising light headedness, minor bleeding, dizziness, or headache. The proportion of subjects with any adverse event did not reportedly differ in acupuncture versus TENS or usual care groups.

#### 3.17.2. Acupuncture—Nonrandomized Studies

In one nonrandomized controlled trial [41], discomfort or soreness in the acupuncture, chiropractic therapy, and massage groups were 5.0%, 8.0%, and 7.0%, respectively.

#### 3.17.3. Manipulation/Mobilization—RCTs

The reported events in RCTs were mostly moderate in severity and of transient nature (e.g., increased pain) [69, 98, 106, 118, 121, 180–184]. In one RCT [121, 185], after 2 weeks of treatment, patients with neck pain receiving manipulation were not at significantly increased risk for having an adverse event compared to patients receiving mobilization (OR = 1.44, 95% CI: 0.83, 2.49). In another RCT [118], the proportion of patients with neck pain having adverse events was similar in manipulation versus Diazepam groups (9.5% versus 11.1%).

#### 3.17.4. Manipulation/Mobilization—Nonrandomized Studies

In two case control studies [186, 187], subjects younger than 45 years of age with vertebro-basilar artery (VBA) stroke were more likely to visit a chiropractic or primary care physician than subjects without VBA stroke. This association was not observed in older subject visiting the chiropractic clinic. In the first case-control study [187], the excess risk of vascular accident was observed for both, subjects undergoing chiropractic care and subjects undergoing primary care treatments. In the second case-control study [186], subjects with cervical artery dissection were more likely to have had spinal manipulation within 30 days (OR = 6.62, 95% CI: 1.4, 30.0). In one cohort study, rate of complications did not differ between subjects with low-back pain receiving manipulation plus mobilization versus no treatment [188]. In another prospective cohort study of 68 subjects with chronic LBP [189], treatment with medication-assisted manipulation or spinal manipulation alone for at least 4 weeks did not lead to any complications requiring institutional review board notification.

#### 3.17.5. Massage

In a few RCTs [76, 142, 147, 190–192], subjects receiving massage experienced worsening of back/neck pain or soreness of mild and transient nature. One study reported allergic reactions (rashes and pimples) in 5 subjects due to massage oil. In one RCT [190], the proportion of patients with neck pain having adverse events in massage group was lower (7.0%) compared to acupuncture (33.0%) or placebo-laser (21.0%).

## 4. Discussion

This paper identified a large amount of evidence on comparative effectiveness of single mode CAM interventions for management of low-back and neck pain in subjects with a wide spectrum of causes of pain. 

The benefits of CAM therapies were limited mostly to immediate and short-term posttreatment periods when compared to inactive treatments (no treatment or placebo). The observed benefits were more consistent for the measures of pain intensity than disability. Trials that applied sham-acupuncture tended to produce negative results (i.e., statistically nonsignificant) compared to trials that applied other types of placebo (e.g., TENS, medication, laser) between acupuncture and placebo groups. One explanation for the beneficial effect of sham acupuncture is the diffuse noxious inhibitory controls (DNIC) where neurons in the dorsal horn of the spinal cord are strongly inhibited when a nociceptive stimulus is applied to any part of the body, distinct from their excitatory receptive fields [193]. Another explanation could be the nonspecific effects of attention and beliefs in a potentially beneficial treatment. 

The results were less consistent regarding comparison of CAM therapies to other active treatments (e.g., other CAM therapy, physiotherapy, pain medication, usual care). The degree of clinical importance for the differences in pooled pain intensity observed between the treatment groups for low-back pain was small (acupuncture versus placebo; mobilization versus physical modalities), medium (acupuncture versus no treatment; massage versus relaxation), or large (acupuncture versus manipulation, in favour of manipulation; massage versus physical modalities). 

Due to the small number of economic evaluations, inconsistent standards of comparison, and substantial heterogeneity as well as different healthcare payment systems used in the countries these trials were conducted, it was not possible to apply these findings globally or to reach clear conclusions about the cost-effectiveness of any of these CAM treatments. Acupuncture was cost-effective relative to usual care or no treatment in subjects with back pain. Evidence for massage and mobilization was insufficient.

We identified 4 systematic reviews of acupuncture: one for LBP [194] and 3 for neck pain [195–198]. The LBP review found either acupuncture being superior (1 trial) or no different from sham acupuncture (3 trials). Although the present paper included a much wider range of trials, its results for neck pain were consistent with those of the three reviews [195, 197, 198] in finding acupuncture moderately more beneficial compared to no treatment or placebo immediately or the short-term after treatment. There were 2 reviews that evaluated manipulation and/or mobilization for acute, subacute, or chronic LBP [199, 200]. The first review [199] found manipulation more beneficial than sham but similar to general practitioner care, physical therapy, or exercise. The other review [200], indicated that manipulation did not differ from NSAIDs but was more beneficial than mobilization, general practitioner care, detuned diathermy, or physical therapy. 

The results are similar across the three systematic reviews with respect to the superiority of manipulation and mobilization compared to no treatment of placebo for the various duration of LBP. The discrepancies lie when comparing manipulation or mobilization to other treatments. One review [199] concludes that manipulation or mobilization is equally effective compared to all other treatments, while the other [200] generally finds manipulation more effective than most other forms of therapy, but mostly in the short-term. In our paper, manipulation and mobilization effectiveness is variable depending on symptom duration, outcome, comparator, whether there is exercise or general practitioner care and followup period. Although this variability can be considered as “inconsistent findings”, the overall evidence suggests that manipulation and mobilization are an effective treatment modality compared to other therapies. The three systematic reviews also differ significantly on definition of SMT: the review by Assendelft et al. [199] lumps spinal manipulation and mobilization together and also allows for cointerventions). The synthesis methods were different, one has more language restrictions [200] and uses best evidence methodology, while the other uses meta-analysis for all included trials and includes patients with leg pain [199]. In addition, they only included RCT published prior to 2002. All these reasons can explain differences in the findings and conclusions.

The findings of this paper regarding the effects of manipulation on neck pain were consistent with those of other reviews [9, 201–203]. While some differences in results between this and other two reviews can be explained by the inclusion criteria and grading of trials, the major results in findings were similar. Two other reviews [204, 205] assessed multimodal interventions (mobilization and manipulation combined with other interventions) and therefore were outside the scope of this review. One Cochrane review [206] found massage to be more beneficial than placebo or no treatment for chronic nonspecific LBP at short or long-term followup. 

One of the strengths of this paper is that it identified a large amount of relevant evidence. The reviewers used systematic, comprehensive, and independent strategies to minimize the risk of bias in searching, identifying, retrieving, screening, abstracting, and appraising the primary studies. The search strategy, not restricted by the language or year of publication, was applied to multiple electronic sources. Further strength of this paper is the inclusion of only those trials from which an effect of a single CAM therapy could be isolated. Moreover, the results of individual trials were stratified by spine region (e.g., low-back, neck), duration of pain (acute, subacute, chronic, mixed, and unknown), and cause of pain (specific or nonspecific). 

This paper has its limitations. The reviewed evidence was of low to moderate grade and inconsistent due to substantial methodological and/or clinical diversity, as well as small sample size of many trials, thereby rendering some between-treatment comparisons inconclusive. The differences in the therapy provider's experience, training, and approaches (e.g., deep or superficial massage, choice of trigger points, needling techniques) may have additionally contributed to heterogeneous results. Evidence for acute, subacute, and mixed specific pain was sparse relative to that for chronic nonspecific pain. Quantitative subgroup analyses exploring the effects of age, gender, race, type of treatment provider, or dose of treatment could not be performed due to lack or insufficient data. Poorly and scarcely reported harms data limited our ability to meaningfully compare rates of adverse events between the treatments. This paper focused on manipulation or mobilization to estimate the efficacy. Results from these studies may not be readily applicable to various combinations of interventions used in today's practice. However, the assessment of a single intervention is the first step in teasing out which therapeutic item is more effective in reducing pain and improving function.

This paper assessed the extent of publication bias using a visual inspection of the funnel plot and the Egger's regression-based technique [23]. Although the visual inspection method is not very reliable, it conveys some general idea as to how symmetrical the dispersion of individual trial effect estimates is around more precise effect [207]. The funnel plot of acupuncture placebo-controlled trials showed some degree of asymmetry which may have arisen due to publication bias. Publication bias, if present, may have led to overestimation of the treatment effect of acupuncture compared to placebo in reducing pain intensity. 

In future, results from long-term large head-to-head trials reporting clinically relevant and validated outcomes are warranted to draw more definitive conclusions regarding benefits and safety of CAM treatments relative to each other or to other active treatments. More research is needed to determine which characteristics of CAM therapies (e.g., mode of administration, length of treatments, number of sessions, and choice of spinal region/points) are useful for what conditions. Future studies should also examine the influence treatment-, care provider-, and population-specific variables on treatment effect estimates. It is clear that strong efforts are needed to improve quality of reporting of primary studies of CAM therapies.

## Figures and Tables

**Table 1:** Risk of bias assessment of RCTs of all interventions for low-back pain and neck pain (total of 131 RCTs).

Quality components	*N* studies	%
Adequate method of randomization	57	43.5%
Adequate method of allocation concealment	41	31.3%
Similarity at baseline regarding the most important prognostic indicators	89	67.9%
Appropriate patient blinding to the intervention	30	22.9%
Appropriate care provider blinding to the intervention	4	3.1%
Appropriate outcome assessor blinding to the intervention	66	50.4%
Similar or no cointerventions between-groups	40	30.5%
Acceptable compliance in all groups	53	40.5%
Described and acceptable drop-out rates	99	75.6%
Similarity of timing of the outcome assessment in all groups	118	90.1%
Inclusion of an intention-to-treat analysis	57	45.5%
Absence of selective outcome reporting	78	59.5%
Absence of other potential bias	7	5.3%

Total risk of bias scores (max 13); median (IQR)	6	4–7

**Table 2:** Summary of findings of acupuncture for low-back pain (only pain and functional outcomes).

Duration and cause of pain	Outcomes	GRADE*	Findings
**Acupuncture versus no treatment**			

Acute/subacute, mixed, and unknown (specific, nonspecific)	NA	*Insufficient * No trial	NA

Chronic nonspecific	Pain intensity score (VAS)	*Moderate * Design: RCT ROB: Medium Consistency: yes Directness: yes Precision: yes	Four trials showed a significant immediate/short-term posttreatment benefit of acupuncture [35, 48, 51, 52]. The pooled estimate was based on 3 trials (short-term posttreatment mean score difference: −1.19, 95% CI: −2.17 to −0.21) [35, 48, 52]. See Figure 3.
	Pain Disability Index	*Moderate * Design: RCT ROB: Low Consistency: NA (only 1 trial) Directness: yes	One trial showed greater improvement in pain disability index with acupuncture (Mean difference: −8.2, 95% CI: −12.0 to −4.4) [51].

Chronic specific	NA	*Insufficient * No trial	NA

**Acupuncture versus placebo**			

Acute/subacute nonspecific	Pain intensity score (VAS)	*Moderate * Design: RCT ROB: Medium Consistency: yes Directness: yes	In two trials [31, 53], short-term posttreatment pain intensity score was not significantly different between acupuncture and placebo groups. Mean score difference: 10.6, 95% CI: −4.1, 25.3^,^Mean score: 49.9 ± 22.2 versus 51.8 ± 26.1, *P* > 0.05).
	Roland-Morris Disability score	*Low * Design: RCT ROB: Medium Consistency: NA (only 1 trial) Directness: yes	In one trial, acupuncture was not significantly different from placebo at 3 months (mean score difference: 2.6, 95% CI: −0.7, 5.9) [53].

Acute/sub acute specific	NA	*Insufficient * No trial	NA

Chronic nonspecific	Pain intensity score (modified MPQ, VAS, von Korff Chronic Pain Grade Scale: 0–10)	*Moderate * Design: RCT ROB: Medium Consistency: yes Directness: yes Precision: yes	Acupuncture was compared to placebo in 16 trials [32, 45, 51, 54–67]. The results of these trials were conflicting. The pooled estimates of 10 trials showed a significant benefit of acupuncture but only immediately posttreatment (mean score difference −0.59, 95% CI: −0.93, −0.25) [51, 55, 56, 58, 59, 61–65, 67]. The mean score differences at short- (−1.11, 95% CI: −2.33, 0.11) [54–56, 58], intermediate- (−0.18, 95% CI: −0.85, 0.49) [51, 54, 67], and long-term (−0.21, 95% CI: −0.64, 0.22) [51, 54, 63, 67] followups after the sessions were not statistically significant. See Figure 4
	Roland-Morris Disability score	*Moderate * Design: RCT ROB: Medium Consistency: yes Directness: yes	The pooled estimate of two trials was not statistically significant (mean score difference*: *0.81, 95% CI: −0.27, 1.9) [62, 67].

Chronic specific	NA	*Insufficient * No trial	NA

Mixed (specific, nonspecific)	NA	NA	NA

Unknown nonspecific	Pain intensity score (VAS)	*Moderate * Design: RCT ROB: Medium Consistency: yes Directness: yes	In one trial [68], there was no significant difference in the proportions of subjects with improved pain (not specified) between the acupuncture versus placebo (sham-acupuncture). Either real needling [30] or total body acupuncture [33] was superior to sham needling in reducing pain intensity immediately posttreatment. For example, in one study [30], the mean pain intensity (VAS score) was 37.3 in acupuncture group and 64.1 in the placebo group.

Unknown specific	NA	*Insufficient * No trial	NA

**Acupuncture versus medication**			

Acute/subacute (specific, nonspecific)	NA	*Insufficient * No trial	NA

Chronic nonspecific	Pain intensity score (VAS)	*Low * Design: RCT ROB: High Consistency: no Directness: yes	There was no significant difference between acupuncture and medication immediately posttreatment. The pooled estimate was based on four trials (mean score difference: 0.11, 95% CI: −1.42, 1.65) [49, 69–71].
	Oswestry Disability Index	*Low * Design: RCT ROB: High Consistency: no Directness: yes	In one trial, [69, 72] acupuncture achieved better score than medication (13 versus 24). The pooled estimate based on two trials showed no significant difference (mean score difference: −2.40, 95% CI: −12.20, 7.40) [69, 70].

Chronic specific	NA	*Insufficient * No trial	NA

Mixed nonspecific	NA	*Insufficient * No trial	NA

Mixed specific	No pain or function outcome reported	—	NR

Unknown nonspecific	No pain or function outcome reported	—	NR

Unknown specific	NA	*Insufficient * No trial	NA

**Acupuncture versus physiotherapy**			

Acute/subacute (specific, nonspecific)		*Insufficient * No trial	

Chronic nonspecific	Oswestry Disability Index	*Low * Design: RCT ROB: Medium Consistency: NA (only 1 trial) Directness: yes	One trial showed manual acupuncture to be significantly superior to physiotherapy (consisted of light, electricity, and/or heat therapy) [26]. Acupuncture group: 38.58 ± 5.0 (before) and 11.55 ± 3.24 (after) Physiotherapy group: 40.24 ± 5.8 (before) and 18.83 ± 5.24 (after).

Chronic specific	NA	*Insufficient * No trial	NA

Mixed/unknown (specific, nonspecific)	NA	*Insufficient * No trial	NA

**Acupuncture versus manipulation**			

Acute/subacute (specific, nonspecific)		*Insufficient * No trial	

Chronic nonspecific	Pain intensity score (VAS)	*Low * Design: RCT ROB: High Consistency: no Directness: yes Precision: yes	There were significant reductions in pain intensity in favour of manipulation (pooled mean difference in VAS score: 3.70, 95% CI: 1.5, 5.8) [69, 70]. See Figure 5.

Chronic specific	NA	*Insufficient * No trial	NA

Mixed/unknown (specific, nonspecific)	NA	*Insufficient * No trial	NA

**Acupuncture versus massage**			

Acute/subacute (specific, nonspecific)	NA	*Insufficient * No trial	NA

Chronic nonspecific	Symptom bothersomeness scale score (0 to 10)	*Low * Design: RCT ROB: Medium Consistency: NA (only 1 trial) Directness: yes	One trial showed massage to be significantly better than manual acupuncture at long-term followup (*P* = 0.002) [36]. Massage group—at baseline: 6.2 (95% CI: 5.8, 6.6) and at 1 year: 3.2 (95% CI: 2.5, 3.9). Acupuncture group—at baseline: 6.2 (95% CI: 5.8, 6.5) and 4.5 (95% CI: 3.8, 5.2).
	Roland-Morris Disability score	*Low * Design: RCT ROB: Medium Consistency: NA (only 1 trial) Directness: yes	One trial showed massage to be significantly better than manual acupuncture at immediate- (*P* = 0.01) or long-term followup (*P* = 0.05) [36]. Mean values at baseline, 4 weeks and 1 year after treatment in the massage group: 11.8 (95% CI: 10.8, 12.7), 7.9 (95% CI: 6.9, 9.0), and 6.8 (95% CI: 5.5, 8.1) [36]. Mean values at baseline, 4 weeks and 1 year after treatment in the acupuncture group: 12.8 (95% CI: 11.7, 13.8), 9.1 (95% CI: 7.8, 9.9) and 8.0 (95% CI: 6.6, 9.3) [36].

Chronic specific	NA	*Insufficient * No trial	NA

Mixed/unknown (specific, nonspecific)	NA	*Insufficient * No trial	NA

**Acupuncture versus usual care**			

Acute/subacute specific	NA	*Insufficient * No trial	NA

Acute/subacute nonspecific	Roland-Morris Disability score	*Low * Design: RCT ROB: Medium Consistency: NA (only 1 trial) Directness: yes	In one trial [41], the addition of acupuncture to usual care did not improve the degree of disability (RMDQ score) compared to usual care alone immediately, shortly, or intermediate-term posttreatment.

Chronic specific	NA	*Insufficient * No trial	NA

Chronic nonspecific	Roland-Morris Disability score	*Moderate * Design: RCT ROB: Medium Consistency: yes Directness: yes	In two trials, subjects who received acupuncture significantly improved in disability compared to subjects in usual care groups at short-term or intermediate-term followup after treatment [47, 67].
	Pain intensity score (VAS)	*Moderate * Design: RCT ROB: Medium Consistency: yes Directness: yes	In two trials, subjects who received acupuncture significantly improved in pain intensity compared to subjects in usual care groups at short-term or intermediate-term followup after treatment [47, 67].

Mixed specific	NA	*Insufficient * No trial	NA

Mixed nonspecific	Disability score (Oswestry)	*Low * Design: RCT ROB: Medium Consistency: NA (only 1 trial) Directness: yes	In one trial [208], a long-term posttreatment disability score was not significantly different between the acupuncture and usual care groups (Oswestry score: *−*3.4, 95% CI: *−*7.8, 1.0).
	Pain intensity score (MPQ)	*Low * Design: RCT ROB: Medium Consistency: NA (only 1 trial) Directness: yes	In one trial [208], a long-term posttreatment pain intensity was not significantly different between the acupuncture and usual care groups (mean difference in MPQ score: *−*0.2, 95% CI: *−*0.6, 0.1).

Unknown (specific, nonspecific)	NA	*Insufficient * No trial	NA

*Precision in formal grading was applied only to pooled results.

VAS: visual analog scale; RMDQ: Roland-Morris disability scale; MPQ: McGill pain questionnaire; PDI: pain disability index; NPQ: neck pain questionnaire; NA: not applicable; ROB: risk of bias; RCT: randomized controlled trial.

**Table 3:** Summary of findings of acupuncture for neck pain (only pain and functional outcomes).

Duration and cause of pain	Outcomes	GRADE*	Findings
**Acupuncture versus no treatment**			

Acute/subacute, chronic, and mixed, (specific, nonspecific)	NA	*Insufficient * No trial	NA

Unknown specific	Pain intensity score (SF-MPQ)	*Low * Design: RCT ROB: Medium Consistency: NA (only 1 trial) Directness: yes	In one trial [75], acupuncture was significantly better than no treatment in reducing pain intensity short-term after the end of treatment (mean change: −15.2 ± 13.3 versus −5.3 ± 8.7, *P* = 0.043).

Unknown nonspecific	NA	*Insufficient * No trial	NA

**Acupuncture versus placebo**			

Acute/subacute specific, nonspecific	NA	*Insufficient * No trial	NA

Chronic specific	Pain intensity score (VAS)	*Moderate * Design: RCT ROB: Medium Consistency: yes Directness: yes Precision: yes	In three trials, acupuncture [77, 209] or dry needling [78] was similar to sham acupuncture [77] or laser acupuncture [78, 209] immediately or at short term after the treatment. In one of these trials [78], posttreatment mean VAS values in dry needling and sham laser acupuncture groups were 29.2 (±21.9) and 28.0 (±19.4), respectively. The meta-analysis of two trials indicated no significant difference between acupuncture and placebo immediately after the end of treatment (pooled mean difference: 0.27, 95% CI: −0.60, 1.13) [79]. See Figure 6.
	NDI score	*Low * Design: RCT ROB: Medium Consistency: NA (only 1 trial) Directness: yes	In one trial [77], the mean disability score was not significantly different between acupuncture and sham-acupuncture groups immediately posttreatment (5.5 ± 4.5 versus 6.2 ± 3.1, *P* = 0.52).

Chronic nonspecific	Pain intensity score (VAS)	*Low * Design: RCT ROB: Medium Consistency: no Directness: yes Precision: yes	The meta-analysis of three trials showed no significant difference between acupuncture and sham-acupuncture immediately posttreatment (pooled mean difference: −0.24, 95% CI: −1.20, 0.73) [80–82] (See Figure 6). Trials comparing acupuncture to other types of placebos (e.g., TENS, drug) [83, 85–87, 210] could not be pooled due to heterogeneity across outcomes, followup periods, or missing data.
	NDI score	*Moderate * Design: RCT ROB: Low Consistency: NA (only 1 trial) Directness: yes	In one trial [83, 210], intermediate posttreatment mean disability was significantly reduced in acupuncture compared to placebo group (8.89 ± 6.57 versus 10.72 ± 9.11, *P* < 0.05).

Mixed specific	Pain intensity score (VAS)	*Low * Design: RCT ROB: High Consistency: NA (only 1 trial) Directness: yes	In one trial [88], there was no significant difference between acupuncture and placebo (laser pen) at intermediate-term posttreatment followup (2.59 ± 2.18 versus 2.89 ± 2.63, *P* > 0.05).

Mixed nonspecific	NA	*Insufficient * No trial	NA

Unknown specific	No pain or disability outcome reported	NA	One trial [27] reporting % subjects without symptoms.

Unknown nonspecific	NA	*Insufficient * No trial	NA

**Acupuncture versus pain medication**			

Acute/subacute, mixed (specific, nonspecific)	NA	*Insufficient * No trial	NA

Chronic specific	Pain intensity score (VAS, SF-MPQ)	*Low * Design: RCT ROB: High Consistency: no Directness: yes	Of the three trials [89–91] comparing acupuncture to medications, in two [89, 90] there was no significant difference between acupuncture and injection of lidocaine [89, 90], lidocaine plus corticoid [90], or botulinum toxin [90] at short-term posttreatment followup. In one of the trials [89], two-week posttreatment mean VAS values were 3.82 ± 2.47 for acupuncture and 3.46 ± 2.47 for lidocaine (*P* > 0.05). In another trial [91], acupuncture was better than NSAIDs immediately after treatment (mean VAS score: 1.87 ± 1.90 versus 4.76 ± 2.05, *P* < 0.05).

Chronic nonspecific	Pain intensity score (VAS)	*Low * Design: RCT ROB: High Consistency: yes Directness: yes	None of three trials comparing acupuncture to medication (e.g., NSAIDs, analgesics) demonstrated significant between-group differences [69, 70, 87]. In one of the trials [69], acupuncture had a better mean score versus pain medication group at immediate (mean VAS score: 4.0 ± 4.4 versus 6.0 ± 4.4) or at intermediate-term followup (mean VAS score: 2.5 versus 4.7) [69, 72].

Unknown specific	Pain intensity score (VAS, SF-MPQ)	*Low * Design: RCT ROB: High Consistency: yes Directness: yes	In two trials [28, 92], acupuncture was significantly more effective than injection of lidocaine in the short-term. In one trial [28], the mean pain scores were 5.71 ± 2.49 versus 6.91 ± 3.22 (*P* < 0.05).

Unknown nonspecific	NA	*Insufficient * No trial	NA

**Acupuncture versus physiotherapy**			

Acute/subacute, chronic, mixed, or unknown (specific, nonspecific)	NA	*Insufficient * No trial	NA

**Acupuncture versus mobilization**			

Acute/subacute, mixed, or unknown (specific, nonspecific)	NA	*Insufficient * No trial	NA

Chronic specific	NA	*Insufficient * No trial	NA

Chronic nonspecific	Pain intensity score (VAS)	*Low * Design: RCT ROB: Medium Consistency: NA (only 1 trial) Directness: yes	In one trial [93], there was no significant difference between acupuncture and standard localized mobilization techniques at short- or intermediate-term posttreatment followup (no numerical data on mean scores were reported).
	Disability (NPQ score)	*Low * Design: RCT ROB: Medium Consistency: NA (only 1 trial) Directness: yes	In one trial [93], there was no significant difference between acupuncture and standard localized mobilization techniques at short- or intermediate-term posttreatment followup (no numerical data on mean scores were reported).

**Acupuncture versus usual care**			

Acute/subacute, mixed, or unknown (specific, nonspecific)	NA	*Insufficient * No trial	NA

Chronic specific	NA	*Insufficient * No trial	NA

Chronic nonspecific	Disability (NPQ score)	*Low * Design: RCT ROB: Medium Consistency: NA (only 1 trial) Directness: yes	In one trial [94], acupuncture was added to general practice care and showed no difference in disability (NPQ) compared to general practice care alone immediately posttreatment (22.73 ± 18.64 versus 25.72 ± 16.29, *P* > 0.05).

**Acupuncture versus manipulation**			

Acute/subacute, mixed, or unknown (specific, nonspecific)	NA	*Insufficient * No trial	NA

Chronic specific	Pain intensity score (VAS)	*Low * Design: RCT ROB: High Consistency: NA (only 1 trial) Directness: yes	In one trial [24], there was no significant difference between acupuncture and spinal manipulation at short-term followup (mean VAS: 4.46 ± 3.11 versus 4.43 ± 2.51).

Chronic nonspecific	Pain intensity score (mean % VAS)	*Low * Design: RCT ROB: High Consistency: no Directness: yes	In one trial [69], acupuncture was better than manipulation in reducing pain intensity at *short-term* followup (50.0% versus 42.0%). In another trial [70], *immediate* posttreatment reduction in pain intensity was significantly greater in manipulation (VAS: 33.0%) versus acupuncture (VAS score % reduction not reported).
	Pain intensity score (VAS)	*Low * Design: RCT ROB: High Consistency: NA (only 1 trial) Directness: yes	In one trial [69, 72], median pain intensity scores in the acupuncture and manipulation groups did not differ at *intermediate-term* followup (VAS median scores: 2.5 versus 2.8, *P* = NR).
	Disability score (NDI)	*Low * Design: RCT ROB: High Consistency: yes Directness: yes	Two trials demonstrated significant superiority of manipulation over acupuncture in improving neck disability. In the first trial [70], median NDI score reduction in neck disability immediately posttreatment was significantly greater in manipulation (−10.0, 95% CI: −14.0, −4.0) than acupuncture group (−6.0, 95% CI: −16.0, 2.0). In the second trial [69], the posttreatment NDI values were significantly more improved in manipulation (median: 22; range: 2–44) than acupuncture group (median: 30; range: 16–47); *P* value not reported.

**Acupuncture versus massage**			

Acute/subacute, mixed, or unknown (specific, nonspecific)	NA	*Insufficient * No trial	NA

Chronic specific	Pain intensity score (VAS)	*Low * Design: RCT ROB: High Consistency: NA (only 1 trial) Directness: yes	In one trial [209], acupuncture was significantly better (VAS score scale: 0–100) compared to massage in a short-term posttreatment followup (mean VAS score change from baseline: 24.22 versus 7.89, *P* = 0.005).

Chronic nonspecific	NA	*Insufficient * No trial	NA

*Precision in formal grading was applied only to pooled results.

VAS: visual analog scale; RMDQ: Roland-Morris disability scale; NHP: Nottingham health profile; MPQ: McGill pain questionnaire; PDI: pain disability index; SF: short form; NPQ: neck pain questionnaire; SF-PQ: short form pain questionnaire; PRI: pain rating index; PPI: present pain intensity; NA: not applicable; NDI: neck disability index.

**Table 4:** Summary of findings of spinal manipulation for low-back pain (only pain and functional outcomes).

Duration and cause of pain	Outcomes	GRADE*	Findings
**Manipulation versus no treatment**			

Acute/subacute nonspecific	Pain intensity score (0 to 5)	*Low * Design: RCT ROB: Medium Consistency: NA (one trial) Directness: yes	In one trial [97], there was a significantly lower immediate posttreatment pain intensity in the manipulation group (change from 2.8 to 1.0; *P* = 0.03) compared to “no treatment” group (change from 2.0 to 2.1, *P* > 0.05).

Acute/subacute specific	NA	*Insufficient * No trial	NA

Mixed nonspecific	Pain intensity score (VAS)	*Low * Design: RCT ROB: High Consistency: NA (one trial) Directness: yes	In one trial [98], manipulation showed significant reduction (from baseline) in immediate/short-term posttreatment pain intensity (VAS: 12.20 versus 10.40, *P* < 0.05), while the “no treatment” group did not experience significant reduction in pain intensity (*P* = 0.10).

Mixed specific	NA	*Insufficient * No trial	NA

Chronic or Unknown (nonspecific and specific)	NA	*Insufficient * No trial	NA

**Manipulation versus placebo**			

Acute/subacute, nonspecific	Pain intensity score (VAS)	*Moderate * Design: RCT ROB: Medium Consistency: yes Directness: yes	Four trials [97, 99, 101, 211] showed significant improvements for manipulation in reducing immediate or short-term posttreatment pain. For example, in one trial [211], manipulation was significantly superior to placebo at short-term followup (four-point VAS; *P* < 0.01). Intermediate-term posttreatment data of the same trial showed no significant difference between the groups. In another trial [101], manipulation showed significantly better immediate-term posttreatment pain intensity (percentage of pain-free subjects: 92.0% versus 25.0%, *P* < 0.01).
	Oswestry Disability Index	*Low * Design: RCT ROB: Medium Consistency: NA (one trial) Directness: yes	One trial [99] showed no between-group differences in the immediate and short-term posttreatment follow-ups.

Acute/subacute specific	NA	*Insufficient * No trial	NA

Chronic nonspecific	Pain intensity score (VAS)	*Low * Design: RCT ROB: Medium Consistency: no Directness: yes	In two trials [102, 211], manipulation was significantly better than placebo. In a third trial [103], the immediate posttreatment pain intensity improved more in the manipulation group (1.3 versus 0.7) and in the short-term posttreatment (2.3 versus0.6). There was a significant change within the manipulation group but not within the placebo group. The *P* value for between-group comparison was not reported and therefore the between-group significant difference was not assumed.
	Oswestry Disability Index	*Low * Design: RCT ROB: Medium Consistency: NA (one trial) Directness: yes	In one trial [102], manipulation was significantly better than placebo immediately posttreatment (9.5 ± 6.3 versus 15.5 ± 10.8, *P* = 0.012), but the difference in the short-term posttreatment was not statistically significant (10.6 ± 11.7 versus 14.0 ± 11.7, *P* = 0.41).

Chronic specific	NA	*Insufficient * No trial	NA

Mixed nonspecific	Pain intensity score (VAS)	*Low * Design: RCT ROB: High Consistency: NA (one trial) Directness: yes	One trial [104] showed that immediate posttreatment improvement was numerically greater in the manipulation group (numerical data not reported, and statistical test results were not provided).

Mixed specific	NA	*Insufficient * No trial	NA

Unknown (specific, nonspecific)	NA	*Insufficient * No trial	NA

**Manipulation versus pain medication**			

Acute/subacute, nonspecific	Pain intensity score (VAS)	*Low * Design: RCT ROB: High Consistency: NA (one trial) Directness: yes	One trial showed a nonsignificant advantage of manipulation at the immediate posttreatment followup [211]. This advantage was not sustained at the short- and intermediate posttreatment followups (numerical data not reported, and statistical test results were not provided).

Acute/subacute specific	NA	*Insufficient * No trial	NA

Chronic nonspecific	Pain intensity score (VAS) Immediate posttreatment	*Low * Design: RCT ROB: High Consistency: yes Directness: yes	Two trials [69, 70] showed significantly greater pain reductions with spinal manipulation. The median (IQR) pain intensity went from 5 (4 to 8) to 3 (0 to 7) (*P* = 0.005) with manipulation, and from 5 (3 to 8) to 5 (2 to 7) (*P* = 0.77) with medication [52]. In the other trial, the change was −2.5 (95% CI: −5.0, −21) in the manipulation group and +0.3 (95% CI: −1.0, 1.7) in the medication group [70].
	Pain intensity (subjective score: 5 = poor, 32 = excellent)	*Low * Design: RCT ROB: High Consistency: NA (one trial) Directness: yes	One trial [211] showed that spinal manipulation was not significantly different from medication. Subjective score with manipulation were 2.6 and 4.3 in the short- and intermediate-term. Subjective score with medication were 2.2 and 4.0 in the short- and intermediate-term. (Statistical test results were not provided).
	Oswestry Disability Index	*Low * Design: RCT ROB: High Consistency: yes Directness: yes	Two trials [69, 70] showed significantly greater mean reduction in disability in the manipulation versus pain medication group immediately after treatment (50% [69] and 30.7% [70]).

Chronic specific	NA	*Insufficient * No trial	NA

Mixed or unknown (specific, nonspecific)	NA	*Insufficient * No trial	NA

**Manipulation versus physiotherapy**			

Acute/subacute, nonspecific	Pain intensity score (VAS)	*Low * Design: RCT ROB: High Consistency: NA (one trial) Directness: yes	One trial [211] showed better scores with manipulation at the immediate-, short- and intermediate posttreatment followups (Numerical data not reported, and statistical test results were not provided).

Acute/subacute, specific	NA	*Insufficient * No trial	NA

Chronic nonspecific	Pain intensity score (VAS)	*Low * Design: RCT ROB: High Consistency: NA (one trial) Directness: yes	One trial [211] showed better scores with physiotherapy versus manipulation at the immediate-, short- and intermediate posttreatment followups (numerical data not reported, and statistical test results were not provided).

Chronic specific	NA	*Insufficient * No trial	NA

Mixed nonspecific	Pain intensity score (11-point pain scale)	*Low * Design: RCT ROB: High Consistency: NA (one trial) Directness: yes	In one trial [105], no significant differences were found in short-term posttreatment effects between manipulation and physiotherapy (McKenzie technique based on diagnoses of derangement, dysfunction or postural syndromes).
	Roland-Morris Disability score	*Low * Design: RCT ROB: High Consistency: NA (one trial) Directness: yes	In one trial [105], there was no significant difference between manipulation and physiotherapy (McKenzie technique based on diagnoses of derangement, dysfunction or postural syndromes) in the short-term posttreatment effects.

Mixed specific	NA	*Insufficient * No trial	NA

Unknown (specific, nonspecific)	NA	*Insufficient * No trial	NA

**Manipulation versus usual care**			

Mixed nonspecific	Pain intensity score (100-mm VAS score)	*Low * Design: RCT ROB: Low Consistency: NA (one trial) Directness: yes	In one trial [106], high or low velocity spinal manipulation was not significantly different from minimal conservative medical care. Mean VAS score difference between high velocity manipulation and usual care was 4.0 (95% CI: −4.0, 12.0), whereas this difference between low velocity manipulation and usual care was 5.8 (95% CI: −2.3 to 14.0).

Roland-Morris Disability score	*Low * Design: RCT ROB: Low Consistency: NA (one trial) Directness: yes	One trial [106] showed that manipulation was significantly more effective than medical care alone in improving disability at immediate, short-, or intermediate-term posttreatment followup. The adjusted RMDQ mean change from baseline in the high and low velocity manipulation and medical care groups were 2.7 (95% CI: 2.0, 3.3), 2.9 (95% CI: 2.2, 3.6), and 1.6 (95% CI: 0.5, 2.8), respectively.

Mixed specific	NA	*Insufficient * No trial	NA

Acute, chronic or unknown (specific, nonspecific)	NA	*Insufficient * No trial	NA

**Manipulation versus massage**			

Acute/subacute nonspecific	Pain intensity score (100-mm VAS)	*Low * Design: RCT ROB: High Consistency: NA (one trial) Directness: yes	In one trial [107], there was no significant difference between manipulation and massage immediately posttreatment (mean difference: −24.1 ± 27 and −17.2 ± 25.1, resp.).

Acute/subacute specific	NA	*Insufficient * No trial	NA

Chronic nonspecific	Pain (duration of pain relief)	*Low * Design: RCT ROB: Medium Consistency: NA (one trial) Directness: no	In one trial [108], manipulation was significantly better than massage immediately—and in the short-term after treatment. The mean (SE) duration of pain relief was 8.01 ± 2.02 with manipulation versus 2.94 ± 0.52 with massage.

Chronic specific	NA	*Insufficient * No trial	NA

Mixed or unknown (specific, nonspecific)	NA	*Insufficient * No trial	NA

*Precision in formal grading was applied only to pooled results.

VAS: visual analog scale; RMDQ: Roland-Morris disability scale; MPQ: McGill pain questionnaire; PDI: pain disability index; NPQ: neck pain questionnaire; NA: not applicable; ROB: risk of bias; RCT: randomized controlled trial.

**Table 5:** Summary of findings of manipulation for neck pain (only pain and functional outcomes).

Duration and cause of pain	Outcomes	GRADE*	Findings
**Manipulation versus no treatment**			

Acute/subacute, chronic, and mixed, (specific, nonspecific)	NA	*Insufficient * No trial	NA

Unknown specific	NA	*Insufficient * No trial	NA

Unknown nonspecific	Pain intensity score (VAS)	*Low * Design: RCT ROB: Medium Consistency: NA (only 1 trial) Directness: yes	In one trial [112], there was no significant difference between manipulation and “no treatment” groups in immediate-term posttreatment pain intensity.

**Manipulation versus placebo**			

Acute/subacute specific	NA	*Insufficient * No trial	NA

Acute/subacute nonspecific	Pain intensity score (VAS)	*Low * Design: RCT ROB: High Consistency: yes Directness: yes	In two trials [113, 114], manipulation was significantly more effective than placebo immediately after treatment. In the first trial [113] ipsilateral manipulation (but not contralateral; *P* = 0.93) was significantly better than placebo ultrasound (mean VAS score: 23.6 ± 18.6 versus 46.5 ± 21.8, *P* = 0.001). In the other trial [114], manipulation was significantly better than placebo (light hand placement on the side of neck without application of any side-different pressure or tension) (numerical data not reported; *P* = 0.01).

Chronic specific	NA	*Insufficient * No trial	NA

Chronic nonspecific	Pain intensity score (VAS)	Moderate Design: RCT ROB: Medium Consistency: yes Directness: yes	In two studies [115, 116], manipulation techniques were significantly better than placebo immediately after treatment. In the first trial [115] cervical osteopathy was better than placebo (sham ultrasound). In the second trial [116] a single thoracic manipulation was significantly better than placebo (hand manoeuvre without high velocity thrust).
	Disability score (NDI)	*Low * Design: RCT ROB: Medium Consistency: NA (only 1 trial) Directness: yes	In one trial [116] a single thoracic manipulation was significantly better than placebo (hand manoeuvre without high velocity thrust).

Mixed (specific, nonspecific)	NA	*Insufficient * No trial	NA

Unknown specific	NA	*Insufficient * No trial	NA

Unknown nonspecific	Pain intensity score (VAS)	*Low * Design: RCT ROB: High Consistency: NA (only 1 trial) Directness: yes	In one trial [117], manipulation was significantly better than placebo immediately after treatment (*P* < 0.001). The mean VAS reductions in manipulation and placebo groups were 15.5 (95% CI: 11.8, 19.2) and 4.2 (95% CI: 1.9, 6.6), respectively.

**Manipulation versus pain medication**			

Acute/subacute, mixed, or unknown (specific, nonspecific)	NA	*Insufficient * No trial	NA

Chronic specific	NA	*Insufficient * No trial	NA

Chronic nonspecific	Pain intensity score (VAS)	*Low * Design: RCT ROB: High Consistency: no Directness: yes	In one trial [118] although both manipulation and medication (Diazepam) groups improved, there was no between-group significant difference at short-term followup after treatment (5.0 ± 3.2 versus 1.8 ± 3.1, *P* = 0.20). In two other trials [69, 70], manipulation was significantly better than medication (e.g., NSAIDs, Celebrex, Vioxx, Paracetamol) at immediate/short-term followup after treatment. In one of these trials [69] the proportion of pain-free patients after the treatment was significantly greater in the manipulation group compared to the medication group (27.3% versus 5.0%, *P* = 0.05).
	Disability score (NDI)	*Low * Design: RCT ROB: High Consistency: yes Directness: yes	In two other trials [69, 70], manipulation was significantly better than medication (e.g., NSAIDs, Celebrex, Vioxx, Paracetamol) at immediate/short-term followup after treatment. In one trial, [69] the median (IQR) values for manipulation and medication groups were 22 [26, 30–33, 35, 36, 41, 45, 47–49, 52–66, 66, 68–72, 75, 77–83, 85, 208–210] versus 42 [26, 27, 30, 33, 36, 41, 47, 49, 66–72, 75, 77–83, 85–91, 208–210], respectively. No between-group *P* value was reported. In the other trial [70] the median (95% CI) changes (from baseline) in manipulation and medication groups were −10.00 (95% CI: −14.0, −4.0) versus 0.0 (95% CI: −14.0, 2.7), respectively (*P* < 0.001).

**Manipulation versus physiotherapy**			

Acute/subacute, chronic, mixed, or unknown (specific, nonspecific)	NA	*Insufficient * No trial	NA

**Manipulation versus mobilization**			

Acute/subacute specific	NA	*Insufficient * No trial	NA

Acute/subacute nonspecific	Pain intensity score (VAS)	*Low * Design: RCT ROB: Medium Consistency: NA (only 1 trial) Directness: yes	In one trial [114], there was no statistically significant difference between manipulation and mobilization immediately after treatment (*P* = 0.16; no other numerical data were reported).

Mixed, specific	NA	*Insufficient * No trial	NA

Mixed, nonspecific	Pain intensity score (VAS)—*immediately after treatment *	*Low * Design: RCT ROB: Medium Consistency: yes Directness: yes	Two trials reported comparison of pain intensity between manipulation and mobilization at immediate followup [119, 120]. In the first trial [120] spinal manipulation was significantly better than mobilization (*P* < 0.001). The mean VAS reductions in manipulation and mobilization groups were 3.5 (95% CI: 3.1, 3.9) and 0.4 (95% CI: 0.2, 0.5), respectively. In the second trial [119], manipulation was significantly better (but at borderline due probably to low study power) than mobilization (mean reduction on NRS-101: −17.3 ± 19.5 versus −10.5 ± 14.8, *P* = 0.05).
	Pain intensity score (VAS)—*intermediate-term after treatment *	*Low * Design: RCT ROB: Medium Consistency: NA (only 1 trial) Directness: yes	In one trial [121] the intermediate-term posttreatment differences between the manipulation and mobilization groups were clinically negligible and statistically nonsignificant (NRS-11: −0.02, 95% CI: −0.69, 0.65) and disability (NDI: 0.46, 95% CI: −0.89, 1.82).
	Disability (NDI score)	*Low * Design: RCT ROB: Medium Consistency: NA (only 1 trial) Directness: yes	In one trial [121] the intermediate-term posttreatment differences between the manipulation and mobilization groups were clinically negligible and statistically nonsignificant (mean difference in NDI score: 0.46, 95% CI: −0.89, 1.82).

Chronic or unknown (specific, nonspecific)	NA	*Insufficient * No trial	NA

**Manipulation versus usual care**			

Acute/subacute, chronic, mixed, or unknown (specific, nonspecific)	NA	*Insufficient * No trial	NA

**Manipulation versus acupuncture (see Table 3 for acupuncture for neck pain)**			

**Manipulation versus massage**			

Acute/subacute, chronic, mixed, or unknown (specific, nonspecific)	NA	*Insufficient * No trial	NA

**Manipulation versus exercise**			

Acute/subacute, chronic, mixed, or unknown (specific, nonspecific)	NA	*Insufficient * No trial	NA

*Precision in formal grading was applied only to pooled results.

VAS: visual analog scale; RMDQ: Roland-Morris disability scale; NHP: Nottingham health profile; MPQ: McGill pain questionnaire; PDI: pain disability index; SF: short form; NPQ: neck pain questionnaire; SF-PQ: short form pain questionnaire; PRI: pain rating index; PPI: present pain intensity; NA: not applicable; NDI: neck disability index; IQR: interquartile range.

**Table 6:** Summary of findings of spinal mobilization for low-back pain (only pain and functional outcomes).

Duration and cause of pain	Outcomes	GRADE*	Findings
**Mobilization versus no treatment**			

Acute/subacute, nonspecific	Pain intensity (MPQ)	*Low * Design: RCT ROB: High Consistency: NA (one trial) Directness: yes	In one trial [122] mobilization group had significantly lower pain intensity immediately posttreatment (*P* = 0.048). No further numerical data was provided.

Acute/subacute specific	NA	*Insufficient * No trial	NA

Chronic nonspecific	Pain intensity score (VAS)	*Low * Design: RCT ROB: Medium Consistency: NA (one trial) Directness: yes	In one trial [34] mobilization (Kaltenborn's wedge assisted posteroanterior) was significantly superior to “no treatment.” Immediate posttreatment mean pain score values were 33.40 for mobilization versus 49.77 for “no treatment” (*P* < 0.001).
	Roland-Morris Disability score	*Low * Design: RCT ROB: Medium Consistency: NA (one trial) Directness: yes	In one trial [34] mobilization (Kaltenborn's wedge assisted posteroanterior) was significantly superior to “no treatment.” Immediate posttreatment mean pain score values were 7.69 for mobilization versus 10.64 for “no treatment” (*P* < 0.003).

Chronic specific	Oswestry Disability Index	*Low * Design: RCT ROB: High Consistency: NA (one trial) Directness: yes	One trial [123] showed no difference between-groups immediately posttreatment in disability index: 5.57 (2.38) with mobilization and 2.19 (1.54) with “no treatment”.

Mixed nonspecific	Pain intensity score (VAS)	*Low * Design: RCT ROB: High Consistency: NA (one trial) Directness: yes	In one trial [124] mobilization did not significantly differ from “no treatment” immediately after treatment. The mean difference in pain (overall %) was *−*24.7 with mobilization and *−*11.1 with no treatment (*F* = 2.63, *P* > 0.05).

Mixed specific	NA	*Insufficient * No trial	NA

Unknown (specific, nonspecific)	NA	*Insufficient * No trial	NA

**Mobilization versus placebo**			

Acute/subacute nonspecific	NA	*Insufficient * No trial	NA

Acute/subacute specific	Pain intensity score (VAS)	*Low * Design: RCT ROB: High Consistency: NA (one trial) Directness: yes	In one trial, [125, 126] of subjects with sacroiliac joint dysfunction (96% women), there was no statistically significant difference immediately posttreatment between mobilization and placebo (no numerical data was reported).

Chronic (specific, nonspecific)	NA	*Insufficient * No trial	NA

Mixed nonspecific	Pain intensity score (VAS)	*Low * Design: RCT ROB: Medium Consistency: NA (one trial) Directness: yes	In one trial, [127] mobilization did not significantly differ from placebo in reducing immediate or short-term posttreatment pain intensity. The mean (SD) pain intensity immediately posttreatment was 4.2 (2.5) with mobilization and 4.3 (2.2) with placebo (*P* = 0.8).

Mixed specific	NA	*Insufficient * No trial	NA

Unknown (specific, nonspecific)	NA	*Insufficient * No trial	NA

**Mobilization versus physiotherapy**			

Acute/subacute (specific, nonspecific)	NA	*Insufficient * No trial	NA

Chronic nonspecific	Pain intensity score (VAS)	*Low * Design: RCT ROB: Medium Consistency: no Directness: yes	The pooled estimate of 2 trials showed a significant benefit of mobilization immediately posttreatment (mean difference in VAS score: −0.50, 95% CI: −0.72, −0.28) [128–130].
	Oswestry Disability Index	Moderate Design: RCT ROB: Medium Consistency: yes Directness: yes	The pooled estimate of 2 trials [128–130] showed a significant benefit of mobilization immediately posttreatment (mean difference in disability score: −4.93, 95% CI: −5.91, −3.96).

Chronic specific	Oswestry Disability Index	*Low * Design: RCT ROB: High Consistency: NA (one trial) Directness: yes	One trial [123] showed no difference between-groups immediately posttreatment in disability index: 5.57 (2.38) with mobilization and 2.55 (1.03) with physiotherapy *(physical modalities including exercise)*.

Mixed nonspecific	Oswestry Disability Index	*Low * Design: RCT ROB: Medium Consistency: NA (one trial) Directness: yes	In one trial [131] there was no difference between mobilization and physiotherapy in disability. Mean change (95% CI) in mobilization group at immediate-, short-term, intermediate-term and long-term posttreatment were 7.0 (3.4, 10.2), 5.1 (1.7, 8.4), 9.4 (6.7, 12.1) and 8.4 (5.2, 11.6), respectively. Mean change (95% CI) in the physiotherapy group at immediate-, short-term, intermediate-term and long-term posttreatment were 2.0 (−1.1, 5.1), 4.0 (1.3, 6.7), 4.7 (1.5, 7.9), and 4.4 (1.2, 7.6), respectively. The between-group difference was statistically significant at intermediate and long-term posttreatment followups only.

Mixed specific	NA	*Insufficient * No trial	NA

Unknown (specific, nonspecific)	NA	*Insufficient * No trial	NA

**Mobilization versus manipulation**			

Acute/subacute (nonspecific)	Roland-Morris Disability score	*Low * Design: RCT ROB: Medium Consistency: NA (one trial) Directness: yes	In one trial, [132] the manipulation group had a significantly better disability score compared to the mobilization group immediately posttreatment. The mean (SD) disability scores were 9.1 (5.3) with manipulation and 3.9 (4.3) with mobilization (*P* < 0.04).

Acute/subacute (specific)	NA	*Insufficient * No trial	NA

Chronic, mixed, unknown (specific, nonspecific)	NA	*Insufficient * No trial	NA

**Mobilization versus massage**			

Acute/subacute (specific, nonspecific)	NA	*Insufficient * No trial	NA

Chronic (nonspecific)	Pain intensity score (VAS)	*Low * Design: RCT ROB: High Consistency: NA (one trial) Directness: yes	In one trial [133], short-term posttreatment pain intensity was slightly but significantly greater in the mobilization group compared to the massage group (3.36 ± 0.25 versus 2.48 ± 0.25, *P* = 0.017).

Chronic (specific)	NA	*Insufficient * No trial	NA

Mixed (specific, nonspecific)	NA	*Insufficient * No trial	NA

Unknown (nonspecific)	NA	*Insufficient * No trial	NA

Unknown (specific)	Pain intensity score (VAS)	*Low * Design: RCT ROB: High Consistency: NA (one trial) Directness: yes	In one trial [25] of subjects with disc protrusion, there was no statistically significant difference in posttreatment pain intensity *between the groups *(5.59 ± 0.80 versus 4.71 ± 0.52, *P* > 0.05).

**Mobilization versus exercise**			

Acute/subacute (specific, nonspecific)	NA	*Insufficient * No trial	NA

Chronic (specific, nonspecific)	NA	*Insufficient * No trial	NA

Mixed nonspecific	Pain intensity score (VAS)	*Low * Design: RCT ROB: High Consistency: NA (one trial) Directness: yes	One trial [134] showed no significant difference between mobilization and exercise in reducing pain immediately after the end of a single treatment. The mean change (SD) was 1.7 (2.1) with mobilization and 1.2 (1.4) with exercise (no significant between-group difference).
	Oswestry Disability Index	*Low * Design: RCT ROB: Medium Consistency: NA (one trial) Directness: yes	Mean change (95% CI) in mobilization group at immediate-, short-term, intermediate-term and long-term posttreatment were 7.0 (3.4, 10.2), 5.1 (1.7, 8.4), 9.4 (6.7, 12.1), and 8.4 (5.2, 11.6), respectively [131]. Mean change (95% CI) in the exercise group at immediate-, short-term, intermediate-term and long-term posttreatment were 3.2 (0.4, 6.1), 2.9 (−0.2, 5.9), 3.5 (0.2, 6.8), and 2.2 (−1.2, 5.7), respectively [131]. Difference between-groups was statistically significant for intermediate and long-term posttreatment followups [131].

Mixed specific	NA	*Insufficient * No trial	NA

Unknown (specific, nonspecific)	NA	*Insufficient * No trial	NA

*Precision in formal grading was applied only to pooled results.

VAS: visual analog scale; RMDQ: Roland-Morris disability scale; MPQ: McGill pain questionnaire; PDI: pain disability index; NPQ: neck pain questionnaire; NA: not applicable; ROB: risk of bias; RCT: randomized controlled trial.

**Table 7:** Summary of findings of mobilization for neck pain (only pain and functional outcomes).

Duration and cause of pain	Outcomes	GRADE*	Findings
**Mobilization versus no treatment**			

Acute/subacute or unknown (specific, nonspecific)	NA	*Insufficient * No trial	NA

Chronic specific	NA	*Insufficient * No trial	NA

Chronic nonspecific	Pain intensity score (VAS)	*Low * Design: RCT ROB: Medium Consistency: NA (only 1 trial) Directness: yes	In one trial [135], mobilization was significantly better than “no treatment” group immediately after treatment (*P* = 0.04); the mean VAS score decrease in mobilization group was from 0.68 ± 0.42 to 0.33 ± 0.02 mm. Corresponding numerical data for “no treatment” group was not reported.

Mixed specific	NA	*Insufficient * No trial	NA

Mixed nonspecific	Pain intensity score (VAS)	*Low * Design: RCT ROB: High Consistency: NA (only 1 trial) Directness: yes	In one study [136], the use of bone-setting resulted in a significantly greater mean VAS reduction compared to “no treatment” immediately (18.5, 95% CI: 12.0, 25.1 versus 4.0, 95% CI: −3.1, 11.1; *P* = 0.002), short- (21.2, 95% CI: 12.7, 29.7 versus 6.2, 95% CI: −1.4, 13.8; *P* = 0.01), and intermediate-term (22.9, 95% CI: 13.1, 32.7 versus 5.4, 95% CI: −1.9, 12.8; *P* = 0.005) after treatment; the between-group difference was not significant (14.2, 95% CI: 5.3, 23.1 versus 5.5, 95% CI: −4.9, 15.5; *P* = 0.2) at long-term followup (1 year posttreatment). Similarly, the proportion of improved subjects (> 50% on VAS) in bone setting group was significantly greater compared to “no treatment” group immediately (*P* = 0.04) and intermediate-term (*P* = 0.002) after treatment. This difference was not statistically significant after one year (*P* = 0.2).

**Mobilization versus placebo**			

Acute/subacute specific	NA	*Insufficient * No trial	NA

Acute/subacute nonspecific	Pain intensity score (VAS)	*Low * Design: RCT ROB: Medium Consistency: NA (only 1 trial) Directness: yes	In one trial [114], mobilization was significantly (numerical data not reported; *P* < 0.01) better than placebo (hand placement without any pressure or tension).

Chronic specific	NA	*Insufficient * No trial	NA

Chronic nonspecific	Pain intensity score (VAS)	*Low * Design: RCT ROB: Medium Consistency: NA (only 1 trial) Directness: yes	In one trial [135], mobilization was not significantly (*P* = 0.09) different from placebo (hand placement without movement of vertebral segment). The mean VAS score decrease in mobilization group was from 0.68 ± 0.42 to 0.33 ± 0.02 mm. Corresponding numerical data for placebo group was not reported.

Mixed or unknown (specific, nonspecific)	NA	*Insufficient * No trial	NA

**Mobilization versus pain medication**			

Acute/subacute, chronic, mixed, or unknown (specific, nonspecific)	NA	*Insufficient * No trial	NA

**Mobilization versus Massage**			

Acute/subacute, mixed, or unknown (specific, nonspecific)	NA	*Insufficient * No trial	NA

Chronic specific	NA	*Insufficient * No trial	NA

Chronic nonspecific	Pain intensity score (VAS)	*Low * Design: RCT ROB: Medium Consistency: NA (only 1 trial) Directness: yes	In one trial [137], bone setting was significantly better than massage at intermediate-term after treatment (mean VAS score: 17.9 ± 18.0 versus 25.4 ± 22.0, *P* < 0.05).
	Disability score (NDI)	*Low * Design: RCT ROB: Medium Consistency: NA (only 1 trial) Directness: yes	In one trial [137], bone setting was significantly better than massage at intermediate-term after treatment (mean NDI score: 11.7 ± 9.0 versus 15.3 ± 10.0, *P* < 0.05).

**Mobilization versus manipulation (see Table 5 for manipulation for neck pain)**			

**Mobilization versus usual care **			

Acute/subacute, chronic or unknown (specific, nonspecific)	NA	*Insufficient * No trial	NA

Mixed specific	NA	*Insufficient * No trial	NA

Mixed nonspecific	Pain intensity score (VAS)	*Low * Design: RCT ROB: High Consistency: NA (only 1 trial) Directness: yes	In one trial [138], spinal mobilization was not significantly different from usual care (counseling and advice on staying active, role of psychosocial factors, self-care such as heat application, home exercises, and ergonomic advice) at intermediate-term posttreatment followup (between-group difference in mean VAS score reduction: 0.5, 95% CI: −0.4, 1.3).
	Disability score (NDI)	*Low * Design: RCT ROB: High Consistency: NA (only 1 trial) Directness: yes	In one trial [138] spinal mobilization was not significantly different from usual care (counseling and advice on staying active, role of psychosocial factors, self-care such as heat application, home exercises, and ergonomic advice) at intermediate-term posttreatment followup (between-group difference in mean NDI score reduction: −0.02, 95% CI: −2.3, 2.3).

**Mobilization versus physiotherapy**			

Acute/subacute or unknown (specific, nonspecific)	NA	*Insufficient * No trial	NA

Chronic specific	NA	*Insufficient * No trial	NA

Chronic nonspecific	Pain intensity score (VAS)	*Low * Design: RCT ROB: Medium Consistency: NA (only 1 trial) Directness: yes	In one trial, [137] bone setting was significantly better than physiotherapy (massage, therapeutic stretching, and exercise therapy) at intermediate-term after treatment (mean VAS score: 17.9 ± 18.0 versus 29.6 ± 23.0, *P* < 0.05).
	Disability score (NDI)	*Low * Design: RCT ROB: Medium Consistency: NA (only 1 trial) Directness: yes	In one trial, [137] bone setting was significantly better than physiotherapy (massage, therapeutic stretching, and exercise therapy) at intermediate-term after treatment (mean NDI score: 11.7 ± 9.0 versus 18.4 ± 10.0, *P* < 0.05).

Mixed specific	NA	*Insufficient * No trial	NA

Mixed nonspecific	Pain intensity score (VAS)	*Low * Design: RCT ROB: High Consistency: NA (only 1 trial) Directness: yes	In one trial [138] spinal mobilization was significantly better than physiotherapy (including specific exercises) at intermediate-term posttreatment followup (between-group difference in mean VAS score reduction: 1.0, 95% CI: 0.1, 1.9).
	Disability score (NDI)	*Low * Design: RCT ROB: High Consistency: NA (only 1 trial) Directness: yes	In one trial [138] spinal mobilization was not significantly different physiotherapy at intermediate-term posttreatment followup (between-group difference in mean NDI score reduction: 1.1, 95% CI: −1.3, 3.4).

**Mobilization versus exercise**			

Acute/subacute, chronic, mixed, or unknown (specific, nonspecific)	NA	*Insufficient * No trial	NA

**Mobilization versus acupuncture**			

Acute/subacute, chronic, mixed, or unknown (specific, nonspecific)	NA	*Insufficient * No trial	NA

*Precision in formal grading was applied only to pooled results.

VAS: visual analog scale; RMDQ: Roland-Morris disability scale; NHP: Nottingham health profile; MPQ: McGill pain questionnaire; PDI: pain disability index; NPQ: neck pain questionnaire; SF-PQ: short form pain questionnaire; PRI: pain rating index; PPI: present pain intensity; NA: not applicable; NDI: neck disability index; IQR: interquartile range.

**Table 8:** Summary of findings of massage for low-back pain (only pain and functional outcomes).

Duration and cause of pain	Outcomes	GRADE*	Findings
**Massage versus no treatment**			

Acute/subacute nonspecific	Pain intensity score (VAS)	*Low * Design: RCT ROB: Medium Consistency: NA (one trial) Directness: yes	One trial showed significant short-term posttreatment benefit with massage (VAS: 37.0 ± 19.0 versus 52.0 ± 21.0, *P* < 0.001) [139].
	Oswestry Disability Index	*Low * Design: RCT ROB: Medium Consistency: NA (one trial) Directness: yes	One trial showed significant short-term posttreatment benefit with massage (Oswestry: 16.0 ± 5.0 versus 31.0 ± 12.0, *P* < 0.001) [139].

Acute/subacute, specific	NA	*Insufficient * No trial	NA

Chronic nonspecific	Pain intensity score (SF-36 pain scale)	*Low * Design: RCT ROB: Medium Consistency: NA (one trial) Directness: yes	In one trial, massage (reflexology) was not significantly different from no treatment immediately posttreatment (mean score: 50.0 ± 25.7 versus 41.8 ± 25.6) and in the intermediate-term followup (mean score: 50.7 ± 27.1 versus 44.4 ± 28.5) [140].
	Oswestry Disability Index	*Low * Design: RCT ROB: Medium Consistency: NA (one trial) Directness: yes	In one trial, massage (reflexology) was not significantly different from no treatment immediately posttreatment (mean score: 29.8 ± 19.6 versus 36.7 ± 19.9) and in the intermediate-term followup (mean score: 29.0 ± 20.2 versus 32.9 ± 17.6) [140].

Chronic specific	NA	*Insufficient * No trial	NA

Mixed/unknown (specific, nonspecific)	NA	*Insufficient * No trial	NA

**Massage versus placebo**			

Acute/subacute, nonspecific	Pain intensity score (VAS, MPQ)	Moderate Design: RCT ROB: Medium Consistency: yes Directness: yes	In two trials massage produced significantly lower immediate and short-term posttreatment pain intensity compared to placebo [139, 141].
	Oswestry Disability Index	*Low * Design: RCT ROB: Medium Consistency: NA (one trial) Directness: yes	In two trials massage produced significantly better disability scores compared to placebo [139, 141].
	Roland-Morris Disability Questionnaire	*Low * Design: RCT ROB: Medium Consistency: NA (one trial) Directness: yes	

Acute/subacute, specific	NA	*Insufficient * No trial	NA

Chronic nonspecific	Pain intensity score (VAS, MPQ)	*Low * Design: RCT ROB: High Consistency: NA (one trial) Directness: yes	In one trial, massage (reflexology) had numerically similar degree of improvement in intermediate-term pain intensity (VAS: 2.2 versus 3.3, MPQ: 6.0 versus 7.5), compared to subjects in the placebo group [142].
	Roland-Morris Disability Questionnaire	*Low * Design: RCT ROB: High Consistency: NA (one trial) Directness: yes	In one trial, massage (reflexology) had numerically similar degree of improvement in intermediate-term disability (RMDQ: 4 versus 3.5) compared to subjects in the placebo group [142].

Chronic specific	NA	*Insufficient * No trial	NA

Mixed/unknown (specific, nonspecific)	NA	*Insufficient * No trial	NA

**Massage versus physiotherapy**			

Acute/subacute, mixed, and unknown (specific, nonspecific)	NA	*Insufficient * No trial	NA

Chronic nonspecific	Pain intensity score (VAS, MPQ)	Moderate Design: RCT ROB: Medium Consistency: yes Directness: yes	The meta-analysis of two trials showed a statistically significant difference in favour of massage over physical therapy in reducing pain intensity immediately posttreatment (pooled mean difference on VAS score: −2.11, 95% CI: −3.15, −1.07) [143, 144].
	Roland-Morris Disability Questionnaire and modified Oswestry Disability Index	*Low * Design: RCT ROB: Medium Consistency: NA (one trial) Directness: yes	The mean total RMDQ score immediately posttreatment was significantly lower in the acupressure group than in the physical therapy group (−3.8, 95% CI: −5.7, −1.9) [143]. The mean total ODI score immediately posttreatment was significantly lower in the acupressure group than in the physical therapy group, (− 5.34, 95% CI: −7.62, −3.05) [143].

Chronic specific	NA	*Insufficient * No trial	NA

**Massage versus relaxation**			

Acute/subacute, mixed, and unknown (specific, nonspecific)	NA	*Insufficient * No trial	NA

Chronic nonspecific	Pain intensity score (VAS)	*Low * Design: RCT ROB: High Consistency: yes Directness: yes	The meta-analysis of two trials showed a significantly lower pain intensity with massage compared to relaxation (mean difference: −1.27, 95% CI: −2.46, −0.08) [145, 146]. A third trial not pooled in the meta-analysis [140] did not demonstrate any significant immediate (or intermediate-term) posttreatment differences in pain (immediate posttreatment score: 50.0 ± 25.7 versus 47.2 ± 26.3).
	Oswestry Disability Index	*Low * Design: RCT ROB: Medium Consistency: NA (one trial) Directness: yes	In one trial, massage (reflexology) was not significantly different from relaxation immediately posttreatment (mean score: 29.8 ± 19.6 versus 33.4 ± 23.3) and in the intermediate-term followup (mean score: 29.0 ± 20.2 versus 31.3 ± 21.1) [140].

Chronic specific	NA	*Insufficient * No trial	NA

**Massage versus usual care**			

Acute/subacute, mixed, and unknown (specific, nonspecific)	NA	*Insufficient * No trial	NA

Chronic nonspecific	Pain intensity score (VAS)	*Low * Design: RCT ROB: Medium Consistency: NA (one trial) Directness: yes	In one trial, there was no significant difference between massage and usual care (prescription by physician and behavioural counselling with practice nurse) measured in the intermediate-term followup, mean change scores −0.41 (95% CI: −0.91, 0.09) and −0.32 (95% CI: −0.66, 0.03) for massage and usual care respectively [147].
	Roland-Morris Disability Questionnaire	*Low * Design: RCT ROB: Medium Consistency: NA (one trial) Directness: yes	In one trial, there was no significant difference between massage and usual care (prescription by physician and behavioural counselling with practice nurse) measured in the intermediate-term followup, mean change score −1.96 (95% CI: −0.74, 3.18) and −0.90 (95% CI: −1.76, 0.04) for massage and usual care respectively [147].

Chronic specific	NA	*Insufficient * No trial	NA

**Massage versus exercise**			

Acute/subacute, nonspecific	Pain intensity score (VAS)	*Low * Design: RCT ROB: Medium Consistency: NA (one trial) Directness: yes	In one trial, comprehensive massage was significantly better than exercise. Mean scores in the massage and exercise group at the immediate posttreatment were 0.44 (95% CI: 0.17, 0.71) versus 1.64 (95% CI: 1.3, 2.0) and short-term posttreatment followups 0.42 (95% CI: 0.17, 0.66) versus 1.33 (95% CI: 0.97, 1.7) respectively [140].
	Roland-Morris Disability Questionnaire	*Low * Design: RCT ROB: Medium Consistency: NA (one trial) Directness: yes	In one trial, comprehensive massage was significantly better than exercise. Mean scores in the massage and exercise group at the immediate posttreatment were 2.36 (95% CI: 1.2, 3.5) versus 6.82 (95% CI: 4.3, 9.3) and short-term posttreatment followups 1.54 (95% CI: 0.69, 2.4) versus 5.71 (95% CI: 3.5, 7.9) respectively [140].

Acute/subacute, specific	NA	*Insufficient * No trial	NA

Chronic nonspecific	Pain intensity score (VAS)	Moderate Design: RCT ROB: Medium Consistency: yes Directness: yes	Two trials showed no significant difference between massage and exercise [29, 147].
	Roland-Morris Disability Questionnaire	*Low * Design: RCT ROB: Medium Consistency: NA (one trial) Directness: yes	One trial showed no significant difference between massage and exercise [147].

Chronic specific	NA	*Insufficient * No trial	NA

Mixed or unknown (specific and nonspecific)	NA	*Insufficient * No trial	NA

*Precision in formal grading was applied only to pooled results.

VAS: visual analog scale; RMDQ: Roland-Morris disability scale; MPQ: McGill pain questionnaire; PDI: pain disability index; NPQ: neck pain questionnaire; NA: not applicable; ROB: risk of bias; RCT: randomized controlled trial.

**Table 9:** Summary of findings of massage for neck pain (only pain and functional outcomes).

Duration and cause of pain	Outcomes	GRADE*	Findings
**Massage versus no treatment**			

Acute/subacute or mixed (specific, nonspecific)	NA	*Insufficient * No trial	NA

Chronic specific	Disability score (NPQ)	*Low * Design: RCT ROB: Medium Consistency: NA (only 1 trial) Directness: yes	In one trial [148] massage was significantly better than “no treatment” immediately after treatment (mean NPQ score: 13.24 ± 11.88 versus 35.64 ± 12.54).

Chronic nonspecific	NA	*Insufficient * No trial	NA

Unknown specific	No pain or disability outcome reported	NA	One trial [149] reporting PPT.

Unknown nonspecific	Pain intensity score (VAS)	*Low * Design: RCT ROB: Medium Consistency: NA (only 1 trial) Directness: yes	In one trial [150] both classical and modified massage techniques (strain/counter-strain) were significantly better than “no treatment” immediately after treatment (*P* < 0.001). There was no significant difference between modified and classical massage (mean difference in VAS score: 0.5, 95% CI: −1.0, 1.1). Classical versus “no treatment” (2.7, 95% CI: 1.6, 3.7). Modified versus “no treatment” (2.6, 95% CI: 1.5, 3.7).

**Massage versus placebo**			

Acute/subacute specific	NA	*Insufficient * No trial	NA

Acute/subacute nonspecific	≥2-point decrease on pain score (NRS-11)	*Low * Design: RCT ROB: Medium Consistency: NA (only 1 trial) Directness: yes	In one trial [151] massage was significantly better than placebo immediately after treatment (OR: 7.4, 95% CI: 1.22, 45.02).

Chronic specific	Pain intensity score (VAS)	*Low * Design: RCT ROB: High Consistency: NA (only 1 trial) Directness: yes	In one trial [209] massage was significantly better than placebo (sham laser) immediately or short-term after treatment (VAS: 7.89 versus 17.28, *P* < 0.05).

Chronic nonspecific	NA	*Insufficient * No trial	NA

Mixed (specific, nonspecific)	NA	*Insufficient * No trial	NA

Unknown specific	NA	*Insufficient * No trial	NA

Unknown nonspecific	No pain or disability outcome reported	NA	One trial [152] reporting PPT.

**Massage versus pain medication**			

Acute/subacute, chronic, mixed, or unknown (specific, nonspecific)	NA	*Insufficient * No trial	NA

**Massage versus mobilization (see Table 7 for mobilization for neck pain)**			

**Massage versus manipulation (see Table 5 for manipulation for neck pain)**			

**Massage versus usual care**			

Acute/subacute, chronic, mixed, or unknown (specific, nonspecific)	NA	*Insufficient * No trial	NA

**Massage versus physiotherapy**			

Acute/subacute, chronic, mixed, or unknown (specific, nonspecific)	NA	*Insufficient * No trial	NA

**Massage versus Exercise**			

Acute/subacute, mixed, or unknown (specific, nonspecific)	NA	*Insufficient * No trial	NA

Chronic specific	Disability score (NPQ)	*Low * Design: RCT ROB: Medium Consistency: NA (only 1 trial) Directness: yes	In one trial [148] massage was significantly better than exercise immediately after treatment (mean NPQ score: 13.24 ± 11.88 versus 20.23 ± 12.06).

Chronic nonspecific	NA	*Insufficient * No trial	NA

**Massage versus acupuncture (see Table 3 for acupuncture for neck pain) **			

*Precision in formal grading was applied only to pooled results.

VAS: visual analog scale; RMDQ: Roland-Morris disability scale; NHP: Nottingham health profile; MPQ: McGill pain questionnaire; PDI: pain disability index; SF: short form; NPQ: neck pain questionnaire; SF-PQ: short form pain questionnaire; PRI: pain rating index; PPI: present pain intensity; NA: not applicable; NDI: neck disability index; IQR: interquartile. range; PPT: pressure pain threshold; OR: odds ratio; 95% CI: ninety-five percent confidence interval.

**Table 10:** Summary of findings of spinal manipulation plus mobilization for low-back pain (only pain and functional outcomes).

Duration and cause of pain	Outcomes	GRADE*	Findings
**Manipulation + mobilization versus placebo**			

Acute/subacute nonspecific	Pain intensity (NRS 0–10)	*Low * Design: RCT ROB: Medium Consistency: NA (one trial) Directness: yes	In one trial [153] there were nonsignificant differences in pain intensity. Immediate posttreatment: −2.0 (95% CI: −0.7, 0.3) versus −0.1 (95% CI: −0.6, 0.4); short-term posttreatment: −0.2 (95% CI: −0.7, 0.3) versus 0.0 (95% CI: −0.5, 0.4).
	Roland-Morris Disability score	*Low * Design: RCT ROB: Medium Consistency: NA (one trial) Directness: yes	In one trial [153] there were nonsignificant differences in disability. Immediate posttreatment: −1.0 (95% CI: −2.0, 0.1) versus −0.7 (95%CI: −1.8, 0.4); short-term: −0.5 (95% CI: −1.7, 0.7) versus −0.1 (95% CI: −1.3, 1.1).

Acute/subacute specific	NA	*Insufficient * No trial	NA

Chronic, mixed, unknown (specific, nonspecific)	NA	*Insufficient * No trial	NA

**Manipulation + mobilization versus physiotherapy**			

Acute/subacute, chronic, unknown (specific, nonspecific)	NA	*Insufficient * No trial	NA

Mixed nonspecific	Pain intensity (NRS 0–10)	*Low * Design: RCT ROB: Medium Consistency: NA (one trial) Directness: yes	In one trial [154] the combination of manipulation and mobilization was associated with significantly greater improvements in intermediate- and long-term posttreatment pain intensity (numerical data not provided).

Mixed specific	NA	*Insufficient * No trial	NA

**Manipulation + mobilization versus usual care**			

Acute/subacute, Chronic (specific, nonspecific)	NA	*Insufficient * No trial	NA

Mixed nonspecific	Pain intensity (VAS score)	*Low * Design: RCT ROB: Medium Consistency: NA (one trial) Directness: yes	In one trial [156], the combination of manipulation and mobilization (with or without physical modalities) was not significantly different from medical care alone or medical care combined with physical modalities immediately posttreatment or in the long-term posttreatment measures of pain.
	Roland-Morris Disability score	*Low * Design: RCT ROB: Medium Consistency: NA (one trial) Directness: yes	In one trial [156], the combination of manipulation and mobilization (with or without physical modalities) was not significantly different from medical care alone or medical care combined with physical modalities immediately posttreatment and in the long-term posttreatment measures of disability.

Mixed specific	NA	*Insufficient * No trial	NA

Unknown nonspecific	Oswestry Disability Index	*Low * Design: RCT ROB: Medium Consistency: NA (one trial) Directness: yes	In one trial [212] subjects receiving manipulation plus mobilization had significantly improved long-term disability. The mean change was 1.03 in the manipulation group versus 0.67 in the hospital outpatient treatment group at short-term posttreatment followup, and 0.94 versus 0.73 at intermediate-term posttreatment followup, respectively.

Unknown specific	NA	*Insufficient * No trial	NA

**Manipulation + mobilization versus exercise**			

Acute/subacute, (specific, nonspecific)	NA	*Insufficient * No trial	NA

Chronic nonspecific	Pain intensity (VAS score)	*Low * Design: RCT ROB: Medium Consistency: NA (one trial) Directness: yes	In one trial [158] the manual therapy group showed significantly greater improvements than the exercise therapy group on pain intensity. The immediate posttreatment means (SD) in the manual therapy and exercise group were: 22 (18.56) and 37 (25.12), respectively. The corresponding means (SD) at short-term posttreatment followup were 22 (19.88) versus 39 (22.53). The corresponding means (SD) at intermediate-term posttreatment followup were 21 (14.58) versus 35 (35.89).
	Oswestry Disability Index	*Low * Design: RCT ROB: Medium Consistency: NA (one trial) Directness: yes	In one trial [158] the manual therapy group showed significantly greater improvements than the exercise therapy group on disability. The posttreatment mean (SD) in the manual therapy versus exercise group were: (a) immediate followup: 18 (13.26) versus 30 (10.77) (b) short-term followup: 18 (11.93) versus 30 (14.36) (c) intermediate-term followup: 17 (13.25) versus 26 (14.36).

Chronic specific	NA	*Insufficient * No trial	NA

Mixed, Unknown (specific, nonspecific)	NA	*Insufficient * No trial	NA

*Precision in formal grading was applied only to pooled results.

VAS: visual analog scale; RMDQ: Roland-Morris disability scale; MPQ: McGill pain questionnaire; PDI: pain disability index; NPQ: neck pain questionnaire; NA: not applicable; ROB: risk of bias; RCT: randomized controlled trial.

**Table 11:** Summary of findings of combination of manipulation and mobilization for neck pain (only pain and functional outcomes).

Duration and cause of pain	Outcomes	GRADE*	Findings
**Manipulation + mobilization versus no treatment**			

Chronic nonspecific	Pain intensity (VAS score)	*Low * Design: RCT ROB: Medium Consistency: NA (one trial) Directness: yes	In one trial, spinal manipulation plus mobilization was significantly better in reducing pain intensity and the frequency of headache than no treatment (*P* < 0.001) [160].

Acute/subacute, mixed, unknown (specific, nonspecific)	NA	*Insufficient * No trial	NA

**Manipulation + mobilization versus placebo**			

Acute/subacute, chronic, or unknown (specific, nonspecific)	NA	*Insufficient * No trial	NA

Mixed specific, nonspecific	NA	*Insufficient * No trial	NA

**Manipulation + mobilization versus usual care**			

Acute/subacute, chronic, or unknown (specific, nonspecific)	NA	*Insufficient * No trial	NA

Mixed specific/nonspecific	NA	*Insufficient * No trial	NA

**Manipulation + mobilization versus physiotherapy**			

Acute/subacute, or unknown (specific, nonspecific)	NA	*Insufficient * No trial	NA

Chronic specific	NA	*Insufficient * No trial	NA

Chronic nonspecific	Pain intensity score (VAS)	*Low * Design: RCT ROB: Medium Consistency: NA (only 1 trial) Directness: yes	In one trial [155], the combination of spinal manipulation and mobilization was significantly better than physiotherapy (exercise, massage, heat, electrotherapy, ultrasound, shortwave diathermy) in reducing pain (mean score improvement: 4.5 versus 4.1, *P* < 0.05). The long-term results (12 months posttreatment) of the same trial [161] were reported for the combined sample of subjects with low-back and neck pain and therefore are not presented in this review.

Mixed specific, nonspecific	NA	*Insufficient * No trial	NA

**Manipulation + mobilization versus exercise**			

Acute/subacute, mixed, or unknown (specific, nonspecific)	NA	*Insufficient * No trial	NA

Chronic specific	NA	*Insufficient * No trial	NA

Chronic nonspecific	Headache frequency (mean number per week)	*Low * Design: RCT ROB: Medium Consistency: NA (only 1 trial) Directness: yes	In one trial [160, 162], spinal manipulation plus mobilization did not significantly differ from exercise (low load endurance exercises aimed to train muscle control of the cervicoscapular region) in reducing headache frequency immediately (2.02 ± 0.24 versus 2.37 ± 0.21, *P* > 0.05) or at intermediate-term posttreatment followup (2.12 ± 0.23 versus 2.52 ± 0.24, *P* > 0.05).
	Pain intensity score (VAS)	*Low * Design: RCT ROB: Medium Consistency: NA (only 1 trial) Directness: yes	In one trial [160, 162] spinal manipulation plus mobilization did not significantly differ from exercise (low load endurance exercises aimed to train muscle control of the cervicoscapular region) in reducing pain intensity immediately (3.37 ± 0.39 versus 3.26 ± 0.38, *P* > 0.05) or at intermediate-term posttreatment followup (2.69 ± 0.32 versus 2.83 ± 0.37, *P* > 0.05).
	Disability score (NPQ)	*Low * Design: RCT ROB: Medium Consistency: NA (only 1 trial) Directness: yes	In one trial [160, 162] spinal manipulation plus mobilization did not significantly differ from exercise (low load endurance exercises aimed to train muscle control of the cervicoscapular region) in reducing disability immediately (mean NPQ score change 12.13 ± 1.80 versus 11.03 ± 2.16, *P* > 0.05) or at intermediate-term posttreatment followup (mean NPQ score change 14.21 ± 1.82 versus 15.66 ± 2.01, *P* > 0.05).

**Manipulation + mobilization versus acupuncture**			

Acute/subacute, chronic, mixed, or unknown (specific, nonspecific)	NA	*Insufficient * No trial	NA

**Manipulation + mobilization versus manipulation**			

Acute/subacute, chronic, mixed, or unknown (specific, nonspecific)	NA	*Insufficient * No trial	NA

**Manipulation + mobilization versus mobilization**			

Acute/subacute, chronic, mixed, or unknown (specific, nonspecific)	NA	*Insufficient * No trial	NA

**Manipulation + mobilization versus medication**			

Acute/subacute, chronic, mixed, or unknown (specific, nonspecific)	NA	*Insufficient * No trial	NA

*Precision in formal grading was applied only to pooled results.

VAS: visual analog scale; RMDQ: Roland-Morris disability scale; NHP: Nottingham health profile; MPQ: McGill pain questionnaire; PDI: pain disability index; min: minute(s); hr(s): hour(s); L: low; M: medium; H: high; pt(s): patient(s); SF: short form; NPQ: neck pain questionnaire; SF-PQ: short form pain questionnaire; PRI: pain rating index; PPI: present pain intensity; NA: not applicable; NDI: neck disability index; IQR: interquartile range; PPT: pressure pain threshold; OR: odds ratio; 95% CI: ninety-five percent confidence interval.

## Appendices

### A. Search Strategies

#### A.1. Ovid MEDLINE(R) 1950 to February Week 1 2010

1 exp Neck/or exp spine/or exp back/or Neck Muscles/or Zygapophyseal Joint/
2 pain/or pain, intractable/or pain, referred/
3 (pain* or ache*).tw.
4 3 or 2
5 4 and 1
6 exp back pain/
7 exp back injuries/
8 (backpain* or backache*).tw.
9 exp spinal injuries/
10 exp spinal diseases/
11 ((disc* or disk*) adj3 (degener* or displace* or prolapse* or hernia* or bulge or protrusion* or extrusion* or sequestration* or disorder* or disease* or rupture* or slipped)).tw.
12 ((stenosis or stenoses) adj3 (lumbar or spine or spines or spinal)).tw.
13 (Spondylolys* or spondylolisthes* or Spondylisthes*).tw.
14 (Discitis or diskitis or Spondylodis*).tw.

15 (osteoporo* adj3 compression fracture*).tw.
16 vertebrogenic pain syndrome*.tw.
17 Sciatica/
18 (Sciatica or ischialgia).tw.
19 (Sciatic adj3 (Neuralgia or Bilateral)).tw.
20 Neck Pain/
21 (cervicalgia or Cervicodynia).tw.
22 ((anterior or posterior) adj3 (cervical pain or cervical ache*)).tw.
23 ((cervicogenic or cervicogenic) adj3 headache*).tw.
24 exp neck injuries/
25 (neckache* or neckpai*).tw.

26 (whiplash* or whip lash* or radiculomyelopath* or radiculo-myelopath*).tw.
27 (neck disorder* adj3 radicul*).tw.
28 (failed back or back surgery syndrome* or FBSS).tw.
29 ((Zygapophyseal or Facet or facets) adj3 (syndrome* or degenerat*)).tw.
30 ((back or neck or spine or spinal or lumbar* or thoracic) adj3 (ache* or aching or pain* or strain*)).tw.
31 (lumbago or dorsalgia).tw.
32 (myofascial adj3 (pain* or ache*)).tw.
33 or/5–32
34 Acupuncture/
35 Acupuncture Therapy/
36 Electroacupuncture/
37 (Acupuncture or acu-puncture or electroacupuncture or electro-acupuncture or electric acupuncture or electric acu-puncture or needling or acupressure or acu-pressure or mox?bustion).tw.
38 exp Manipulation, Spinal/
39 Manipulation, Chiropractic/
40 Chiropractic/
41 ((back or neck or spine or spinal or lumbar or cervical or chiropractic* or musculoskeletal* or musculo-skeletal*) adj3 (adjust* or manipulat* or mobiliz* or mobilis*)).tw.
42 (Manual adj therap*).tw.
43 (Manipulati* adj (therap* or medicine)).tw.
44 exp Massage/
45 (massag* or reflexolog* or rolfing or zone therap*).tw.
46 (Chih Ya or Shiatsu or Shiatzu or Zhi Ya).tw.
47 (Flexion adj2 distraction*).tw.
48 (myofascial adj3 (release or therap*)).tw.
49 Muscle energy technique*.tw.
50 Trigger point*.tw.
51 Proprioceptive Neuromuscular Facilitation*.tw.
52 Cyriax Friction.tw.
53 (Lomilomi or lomi-lomi or trager).tw.
54 Aston patterning.tw.
55 (Strain adj counterstrain).tw.
56 Alexander technique*.tw.
57 (Craniosacral Therap* or Cranio-sacral Therap*).tw.
58 (amma or ammo or Effleurage or Petrissage or hacking or Tapotment).tw.
59 Complementary Therapies/
60 ((complement* or alternat* or osteopathic*) adj (therap* or medicine)).tw.
61 (Tui Na or Tuina).tw.
62 or/34–61
63 33 and 62

*The following filters were applied and overlap removed:*

##### A.1.1. Randomized/Controlled Clinical Trials

64 exp Randomized Controlled Trials as topic/
65 Randomized Controlled Trial.pt.
66 Controlled Clinical Trial.pt.
67 (random* or sham or placebo*).tw.
68 placebos/
69 Random Allocation/
70 Single Blind Method/
71 Double Blind Method/
72 ((singl* or doubl* or tripl* or trebl*) adj (blind* or dumm* or mask*)).tw.
73 (RCT or RCTs).tw.
74 (control* adj2 (study or studies or trial*)).tw.
75 or/64–74
76 63 and 75
77 animal/
78 human/
79 77 not 77 and 78
80 76 not 79

##### A.1.2. Systematic Review

81 Meta-Analysis/
82 exp Meta-Analysis as Topic/
83 Meta analysis.pt.
84 (meta analy* or metaanaly* or met analy* or metanaly*).tw.
85 Review Literature as Topic/
86 (collaborative research or collaborative review* or collaborative overview*).tw.
87 (integrative research or integrative review* or integrative overview*).tw.
88 (quantitative adj3 (research or review* or overview*)).tw.
89 (research integration or research overview*).tw.
90 (systematic* adj3 (review* or overview*)).tw.
91 (methodologic* adj3 (review* or overview*)).tw.
92 exp Technology Assessment, Biomedical/
93 (hta or htas or technology assessment*).tw.
94 ((hand adj2 search*) or (manual* adj search*)).tw.
95 ((electronic adj database*) or (bibliographic* adj database*)).tw.
96 ((data adj2 abstract*) or (data adj2 extract*)).tw.
97 (Data adj3 (pool or pooled or pooling)).tw.(5850)
98 (Analys* adj3 (pool or pooled or pooling)).tw.
99 Mantel Haenszel.tw.
100 (Cochrane or PubMed or MEDLINE or EMBASE or PsycINFO or PsycLIT or PsychINFO or PsychLIT or CINAHL or Science Citation Index).ab.
101 or/81–100
102 63 and 101
103 102 not 79
104 103 not 80

##### A.1.3. Safety

81 (ae or to or po or co).fs.
82 (safe or safety or unsafe).tw.
83 (side effect* or side event*).tw.
84 ((adverse or undesirable or harm* or injurious or serious or toxic) adj3 (effect* or reaction* or event* or incident* or outcome*)).tw.
85 (abnormalit* or toxicit* or complication* or consequence* or noxious or tolerabilit*).tw.
86 or/81–85
87 63 and 86
88 87 not 79
89 88 not 80

##### A.1.4. Economics

90 economics/
91 exp “costs and cost analysis”/
92 Value of Life/
93 economics medical/
94 (econom* or cost or costs or costly or costing or price or prices or pricing).ti,ab.
95 (expenditure* not energy).ti,ab.
96 (value adj2 money).ti,ab.
97 budget.ti,ab.
98 or/90–97
99 63 and 98
100 99 not 79
101 100 not (80 or 89)

#### A.2. EMBASE 1980 to 2009 Week 38

1 exp Neck/or exp spine/or exp back/or Neck Muscle/or Back Muscle/or Zygapophyseal Joint/
2 Pain/or Intractable Pain/or Referred Pain/
3 (pain* or ache*).tw.
4 2 or 3
5 1 and 4
6 exp Backache/
7 (backache or backpain).tw.
8 exp Spine Injury/
9 exp Spine Disease/
10 ((disc* or disk*) adj3 (degener* or displace* or prolapse* or hernia* or bulge or protrusion* or extrusion* or sequestration* or disorder* or disease* or rupture* or slipped)).tw.
11 ((stenosis or stenoses) adj3 (lumbar or spine or spines or spinal)).tw.
12 (Spondylolys* or spondylolisthes* or Spondylisthes*).tw.
13 (Discitis or diskitis or Spondylodis*).tw.
14 (osteoporo* adj3 compression fracture*).tw.
15 vertebrogenic pain syndrome*.tw.
16 Ischialgia/
17 (Ischialgia or sciatica).tw.
18 (Sciatic adj3 (Neuralgia or Bilateral)).tw.
19 Neck Pain/
20 (cervicalgia or Cervicodynia).tw.
21 ((anterior or posterior) adj3 (cervical pain or cervical ache*)).tw.
22 ((cervicogenic or cervicogenic) adj3 headache*).tw.
23 exp neck injuries/
24 (neckache* or neckpain*).tw.
25 (whiplash* or whip lash* or radiculomyelopath* or radiculo-myelopath*).tw.
26 (failed back or back surgery syndrome* or FBSS).tw.
27 (myofascial adj3 (pain* or ache*)).tw.
28 ((Zygapophyseal or Facet or facets) adj3 (syndrome* or degenerat*)).tw.
29 ((back or neck or spine or spinal or lumbar* or thoracic) adj3 (ache* or aching or pain* or strain*)).tw.
30 (lumbago or dorsalgia).tw.
31 (neck disorder* adj3 radicul*).tw.
32 or/5–31
33 exp Acupuncture/
34 Electroacupuncture/
35 (Acupuncture or acu-puncture or electroacupuncture or electro-acupuncture or electric* acupuncture or electric* acu-puncture or needling or acupressure or acu-pressure or mox?bustion).tw.
36 exp Manipulative Medicine/
37 chiropractic/
38 ((back or neck or spine or spinal or lumbar or cervical or chiropractic* or musculoskeletal* or musculo-skeletal*) adj3 (adjust* or manipulat* or mobiliz* or mobilis*)).tw.
39 (Manual adj therap*).tw.
40 (Manipulati* adj (therap* or medicine)).tw.
41 Massage/
42 (massag* or reflexolog* or rolfing or zone therap*).tw.
43 (Chih Ya or Shiatsu or Shiatzu or Zhi Ya).tw.
44 (Flexion adj2 distraction*).tw.
45 (myofascial adj3 (release or therap*)).tw.
46 Muscle energy technique*.tw.
47 Trigger point*.tw.
48 Proprioceptive Neuromuscular Facilitation*.tw.
49 Cyriax Friction.tw.
50 (Lomilomi or lomi-lomi or trager).tw.
51 Aston patterning.tw.
52 (Strain adj counterstrain).tw.
53 Alexander technique*.tw.
54 (Craniosacral Therap* or Cranio-sacral Therap*).tw.
55 (amma or ammo or Effleurage or Petrissage or hacking or Tapotment).tw.
56 Alternative Medicine/
57 ((complement* or alternat* or osteopathic*) adj (therap* or medicine)).tw.
58 (Tui Na or Tuina).tw.
59 or/33–58
60 32 and 59

*The following filters were applied and overlap removed:*

##### A.2.1. Randomized/Controlled Clinical Trials

61 Randomized Controlled Trial/
62 exp Controlled Clinical Trial/
63 (random* or sham or placebo*).tw.
64 placebo/
65 Randomization/
66 Single Blind Procedure/
67 Double Blind Procedure/
68 ((singl* or doubl* or tripl* or trebl*) adj (blind* or dumm* or mask*)).tw.
69 (RCT or RCTs).tw.
70 (control* adj2 (study or studies or trial*)).tw.
71 or/61–70
72 60 and 71
73 human.sh.
74 nonhuman.sh.
75 animal.sh.
76 animal experiment.sh.
77 or/74–76
78 77 not (73 and 77)
79 72 not 78

##### A.2.2. Systematic Review

80 Meta Analysis/(34242)
81 “systematic review”/(24457)
82 (meta analy* or metaanaly* or met analy* or metanaly*).tw.(22067)
83 (collaborative research or collaborative review* or collaborative overview*).tw.(834)
84 (integrative research or integrative review* or integrative overview*).tw.(128)
85 (quantitative adj3 (research or review* or overview*)).tw.(1551)
86 (research integration or research overview*).tw.(59)
87 (systematic* adj3 (review* or overview*)).tw.(17008)
88 (methodologic* adj3 (review* or overview*)).tw.(1013)
89 biomedical technology assessment/(5472)
90 (hta or htas or technology assessment*).tw.(1902)
91 ((hand adj2 search*) or (manual* adj search*)).tw.(2396)
92 ((electronic adj database*) or (bibliographic* adj database*)).tw.(2660)
93 ((data adj2 abstract*) or (data adj2 extract*)).tw.(11462)
94 (Data adj3 (pool or pooled or pooling)).tw.(4432)
95 (Analys* adj3 (pool or pooled or pooling)).tw.(3135)
96 Mantel Haenszel.tw.(1463)
97 (Cochrane or PubMed or MEDLINE or EMBASE or PsycINFO or PsycLIT or PsychINFO or PsychLIT or CINAHL or Science Citation Index).ab.(28709)
98 or/80–97(100019)
99 60 and 98(421)
100 99 not 78(421)
101 100 not 79(178)

##### A.2.3. Safety

80 (ae or co or si or to).fs.
81 (safe or safety or unsafe).tw.
82 (side effect* or side event*).tw.
83 ((adverse or undesirable or harm* or injurious or serious or toxic) adj3 (effect* or reaction* or event* or incident* or outcome*)).tw.
84 (abnormalit* or toxicit* or complication* or consequence* or noxious or tolerabilit*).tw.
85 or/80–84
86 60 and 85
87 86 not 78
88 87 not 79

##### A.2.4. Economics

89 health-economics/
90 exp economic-evaluation/
91 exp health-care-cost/
92 (econom* or cost or costs or costly or costing or price or prices or pricing).ti,ab.
93 (expenditure* not energy).ti,ab.
94 (value adj2 money).ti,ab.
95 budget*.ti,ab.
96 socioeconomics/
97 or/89–96
98 60 and 97
99 98 not 78
100 99 not (79 or 88)

#### A.3. AMED <1985 to August 2009>

1 exp Neck/or exp spine/or exp back/or Neck Muscles/
2 pain/or pain intractable/
3 (pain* or ache*).tw.
4 2 or 3
5 1 and 4
6 exp backache/
7 back injuries/
8 (backache* or backpain*).tw.
9 spinal injuries/
10 exp spinal disease/
11 ((disc* or disk*) adj3 (degener* or displace* or prolapse* or hernia* or bulge or protrusion* or extrusion* or sequestration* or disorder* or disease* or rupture* or slipped)).tw.
12 ((stenosis or stenoses) adj3 (lumbar or spine or spines or spinal)).tw.
13 (Spondylolys* or spondylolisthes* or Spondylisthes*).tw.
14 (Discitis or diskitis or Spondylodis*).tw.
15 (osteoporo* adj3 compression fracture*).tw.
16 vertebrogenic pain syndrome*.tw.
17 sciatica/
18 (Sciatica or Ischialgia).tw.
19 (Sciatic adj3 (Neuralgia or Bilateral)).tw.
20 neck pain/
21 (cervicalgia or Cervicodynia).tw.
22 ((anterior or posterior) adj3 (cervical pain or cervical ache*)).tw.
23 ((cervicogenic or cervicogenic) adj3 headache*).tw.
24 exp neck injuries/
25 (neckache* or neckpain*).tw.
26 (neck disorder* adj3 radicul*).tw.
27 (whiplash* or whip lash* or radiculomyelopath* or radiculo-myelopath*).tw.
28 (failed back or back surgery syndrome*).tw.
29 FBSS.tw.
30 ((Zygapophyseal or Facet or facets) adj3 (syndrome* or degenerat*)).tw.
31 ((back or neck or spine or spinal or lumbar* or thoracic) adj3 (ache* or aching or pain* or strain*)).tw.
32 (lumbago or dorsalgia).tw.
33 (myofascial adj3 (pain* or ache*)).tw.
34 or/5–33
35 exp acupuncture/
36 exp acupuncture therapy/
37 (Acupuncture or acu-puncture or electroacupuncture or electro-acupuncture or electric acupuncture or electric acu-puncture or needling or acupressure or acu-pressure or mox?bustion).tw.
38 spinal manipulation/
39 exp manipulation chiropractic/
40 chiropractic/
41 ((back or neck or spine or spinal or lumbar or cervical or chiropractic* or musculoskeletal* or musculo-skeletal*) adj3 (adjust* or manipulat* or mobiliz* or mobilis*)).tw.
42 (Manual adj therap*).tw.
43 (Manipulati* adj (therap* or medicine)).tw.
44 massage/
45 (massag* or reflexolog* or rolfing or zone therap*).tw.
46 (Chih Ya or Shiatsu or Shiatzu or Zhi Ya).tw.
47 (Flexion adj2 distraction*).tw.
48 (myofascial adj3 (release or therap*)).tw.
49 Muscle energy technique*.tw.
50 Trigger point*.tw.
51 Proprioceptive Neuromuscular Facilitation*.tw.
52 Cyriax Friction.tw.
53 (Lomilomi or lomi-lomi or trager or Tui Na or Tuina).tw.
54 Aston patterning.tw.
55 (Strain adj counterstrain).tw.
56 Alexander technique*.tw.
57 (Craniosacral Therap* or Cranio-sacral Therap*).tw.
58 (amma or ammo or Effleurage or Petrissage or hacking or Tapotment).tw.
59 complementary therapies/
60 ((complement* or alternat* or osteopathic*) adj (therap* or medicine)).tw.
61 or/35–60
62 34 and 61

*The following filters were applied and overlap removed:*

##### A.3.1. Randomized/Controlled Clinical Trials

63 randomized controlled trials/
64 randomized controlled trial.pt.
65 controlled clinical trial.pt.
66 (random* or sham or placebo*).tw.
67 placebos/
68 double blind method/or random allocation/
69 ((singl* or doubl* or tripl* or trebl*) adj (blind* or dumm* or mask*)).tw.
70 (RCT or RCTs).tw.
71 (control* adj2 (study or studies or trial*)).tw.
72 randomised controlled trial.pt.
73 or/63–72
74 62 and 73

##### A.3.2. Systematic Review

75 meta analysis/
76 meta analysis.pt.
77 (meta analy* or metaanaly* or met analy* or metanaly*).tw.
78 (collaborative research or collaborative review* or collaborative overview*).tw.
79 (integrative research or integrative review* or integrative overview*).tw.
80 (quantitative adj3 (research or review* or overview*)).tw.
81 (research integration or research overview*).tw.
82 (systematic* adj3 (review* or overview*)).tw.
83 (methodologic* adj3 (review* or overview*)).tw.
84 (hta or htas or technology assessment*).tw.
85 ((hand adj2 search*) or (manual* adj search*)).tw.
86 ((electronic adj database*) or (bibliographic* adj database*)).tw.
87 ((data adj2 abstract*) or (data adj2 extract*)).tw.
88 (Data adj3 (pool or pooled or pooling)).tw.
89 (Analys* adj3 (pool or pooled or pooling)).tw.
90 Mantel Haenszel.tw.
91 (Cochrane or PubMed or MEDLINE or EMBASE or PsycINFO or PsycLIT or PsychINFO or PsychLIT or CINAHL or Science Citation Index).ab.
92 or/75–91(2843)
93 62 and 92(150)
94 93 not 74.

##### A.3.3. Safety

75 (safe or safety or unsafe).tw.
76 (side effect* or side event*).tw.
77 ((adverse or undesirable or harm* or injurious or serious or toxic) adj3 (effect* or reaction* or event* or incident* or outcome*)).tw.
78 (abnormalit* or toxicit* or complication* or consequence* or noxious or tolerabilit*).tw.
79 adverse effects/
80 or/75–79
81 62 and 80
82 81 not 74

##### A.3.4. Economics

84 Economics/
85 exp “costs and cost analysis”/or patient satisfaction/or “quality of life”/
86 (econom* or cost or costs or costly or costing or price or prices or pricing or budget*).ti,ab.
87 (expenditure* not energy).ti,ab.
88 (value adj2 money).ti,ab.
89 (QOL or QOLY or QOLYs or HRQOL or QALY or QALYs).ti,ab.
90 or/84–89
91 62 and 90
92 91 not (74 or 82)

#### A.4. ACP Journal Club <1991 to August 2008>

1 (backpain* or backache*).tw.
2 ((disc* or disk*) adj3 (degener* or displace* or prolapse* or hernia* or bulge or protrusion* or extrusion* or sequestration* or disorder* or disease* or rupture* or slipped)).tw.
3 ((stenosis or stenoses) adj3 (lumbar or spine or spines or spinal)).tw.
4 (Spondylolys* or spondylolisthes* or Spondylisthes*).tw.
5 (Discitis or diskitis or Spondylodis*).tw.
6 (osteoporo* adj3 compression fracture*).tw.
7 vertebrogenic pain syndrome*.tw.
8 (Sciatica or ischialgia).tw.
9 (Sciatic adj3 (Neuralgia or Bilateral)).tw.
10 (cervicalgia or Cervicodynia).tw.
11 ((anterior or posterior) adj3 (cervical pain or cervical ache*)).tw.
12 ((cervicogenic or cervicogenic) adj3 headache*).tw.
13 (neckache* or neckpain*).tw.
14 (whiplash* or whip lash* or radiculomyelopath* or radiculo-myelopath*).tw.
15 (failed back or back surgery syndrome* or FBSS).tw.
16 ((Zygapophyseal or Facet or facets) adj3 (syndrome* or degenerat*)).tw.
17 ((back or neck or spine or spinal or lumbar* or thoracic) adj3 (ache* or aching or pain* or strain*)).tw.
18 (lumbago or dorsalgia).tw.
19 (myofascial adj3 (pain* or ache*)).tw.
20 (neck disorder* adj3 radicul*).tw.
21 or/1–20
22 (Acupuncture or acu-puncture or electroacupuncture or electro-acupuncture or electric acupuncture or electric acu-puncture or needling or acupressure or acu-pressure or mox?bustion).tw.
23 ((back or neck or spine or spinal or lumbar or cervical or chiropractic* or musculoskeletal* or musculo-skeletal*) adj3 (adjust* or manipulat* or mobiliz* or mobilis*)).tw.
24 (Manual adj therap*).tw.
25 (Manipulati* adj (therap* or medicine)).tw.
26 (massag* or reflexolog* or rolfing or zone therap*).tw.
27 (Chih Ya or Shiatsu or Shiatzu or Zhi Ya).tw.
28 (Flexion adj2 distraction*).tw.
29 (myofascial adj3 (release or therap*)).tw.
30 Muscle energy technique*.tw.
31 Trigger point*.tw.
32 Proprioceptive Neuromuscular Facilitation*.tw.
33 Cyriax Friction.tw.
34 (Lomilomi or lomi-lomi or trager or Tui Na or Tuina).tw.
35 Aston patterning.tw.
36 (Strain adj counterstrain).tw.
37 Alexander technique*.tw.
38 (Craniosacral Therap* or Cranio-sacral Therap*).tw.
39 (amma or ammo or Effleurage or Petrissage or hacking or Tapotment).tw.
40 ((complement* or alternat* or osteopathic*) adj (therap* or medicine)).tw.
41 or/22–40
42 21 and 41

#### A.5. CINAHL <1982 to September Week 3 2008>

1 Neck/
2 Back/
3 exp Spine/
4 Neck Muscles/
5 or/1–4
6 pain/
7 Referred Pain/
8 (pain* or ache*).tw.
9 or/6–8
10 5 and 9
11 exp Back Pain/
12 exp Back Injuries/
13 (backache* or backpain*).tw.
14 exp Spinal Injuries/
15 exp Spinal Diseases/
16 ((disc* or disk*) adj3 (degener* or displace* or prolapse* or hernia* or bulge or protrusion* or extrusion* or sequestration* or disorder* or disease* or rupture* or slipped)).tw.
17 ((stenosis or stenoses) adj3 (lumbar or spine or spines or spinal)).tw.
18 (Spondylolys* or spondylolisthes* or Spondylisthes*).tw.
19 (Discitis or diskitis or Spondylodis*).tw.
20 (osteoporo* adj3 compression fracture*).tw.
21 vertebrogenic pain syndrome*.tw.
22 Sciatica/
23 (Sciatica or Ischialgia).tw.
24 (Sciatic adj3 (Neuralgia or Bilateral)).tw.
25 Neck Pain/
26 (cervicalgia or Cervicodynia).tw.
27 ((anterior or posterior) adj3 (cervical pain* or cervical ache*)).tw.
28 ((cervicogenic or cervicogenic) adj3 headache*).tw.
29 exp Neck Injuries/
30 (neckache* or neckpain*).tw.
31 (whiplash* or whip lash*).tw.
32 (failed back or back surgery syndrome* or FBSS).tw.
33 (neck disorder* adj3 radicul*).tw.
34 ((Zygapophyseal or Facet or facets) adj3 (syndrome* or degenerat*)).tw.
35 ((back or neck or spine or spinal or lumbar* or thoracic) adj3 (ache* or aching or pain* or strain*)).tw.
36 (lumbago or dorsalgia).tw.
37 (myofascial adj3 (pain* or ache*)).tw.
38 or/10–37
39 exp Acupuncture/
40 (Acupuncture or acu-puncture or electroacupuncture or electro-acupuncture or electric* acupuncture or electric* acu-puncture or acupressure or acu-pressure or mox?bustion).tw.
41 exp chiropractic/or manipulation, chiropractic/
42 ((back or neck or spine or spinal or lumbar or cervical or chiropractic* or musculoskeletal* or musculo-skeletal*) adj3 (adjust* or manipulat* or mobiliz* or mobilis*)).tw.
43 (Manual adj therap*).tw.
44 (Manipulati* adj (therap* or medicine)).tw.
45 exp Massage/
46 (massag* or reflexolog* or rolfing or zone therap*).tw.
47 (Chih Ya or Shiatsu or Shiatzu or Zhi Ya or Tui Na).tw.
48 (Flexion adj2 distraction*).tw.
49 (myofascial adj3 (release or therap*)).tw.
50 Muscle energy technique*.tw.
51 Trigger point*.tw.
52 Proprioceptive Neuromuscular Facilitation*.tw.
53 Cyriax Friction.tw.
54 (Lomilomi or lomi-lomi or trager or Tui Na or Tuina).tw.
55 Aston patterning.tw.
56 (Strain adj counterstrain).tw.
57 Alexander technique*.tw.
58 (Craniosacral Therap* or Cranio-sacral Therap*).tw.
59 (amma or ammo or Effleurage or Petrissage or hacking or Tapotment).tw.
60 Alternative Therapies/
61 ((complement* or alternat* or osteopathic*) adj (therap* or medicine)).tw.
62 or/39–61
63 38 and 62

*The following filters were applied and overlap removed:*

##### A.5.1. Randomized/Controlled Clinical Trials

64 exp Clinical Trials/
65 clinical trial.pt.
66 (random* or sham or placebo*).tw.
67 placebos/
68 Random Assignment/
69 ((singl* or doubl* or tripl* or trebl*) adj (blind* or dumm* or mask*)).tw.
70 (RCT or RCTs).tw.
71 (control* adj2 (study or studies or trial*)).tw.
72 or/64–71
73 63 and 72

##### A.5.2. Systematic Review

74 systematic review.pt.
75 Meta Analysis/
76 (meta analy* or metaanaly* or met analy* or metanaly*).tw.
77 (collaborative research or collaborative review* or collaborative overview*).tw.
78 (integrative research or integrative review* or integrative overview*).tw.
79 (quantitative adj3 (research or review* or overview*)).tw.
80 (integrative research or research integration or research overview*).tw.
81 (systematic* adj3 (review* or overview*)).tw.
82 (methodologic* adj3 (review* or overview*)).tw.
83 (hta or htas or technology assessment*).tw.
84 ((hand adj2 search*) or (manual* adj2 search*)).tw.
85 ((electronic adj database*) or (bibliographic* adj database*)).tw.
86 ((data adj2 abstract*) or (data adj2 extract*)).tw.
87 (data adj3 (pool or pooled or pooling)).tw.
88 (analys* adj3 (pool or pooled or pooling)).tw.
89 Mantel Haenszel.tw.
90 (Cochrane or PubMed or MEDLINE or EMBASE or PsycINFO or PsycLIT or PsychINFO or PsychLIT or CINAHL or Science Citation Index).ab.
91 or/74–90
92 63 and 91
93 92 not 73

##### A.5.3. Safety

74 (safe or safety or unsafe).tw.
75 (side effect* or side event*).tw.
76 ((adverse or undesirable or harm* or injurious or serious or toxic) adj3 (effect* or reaction* or event* or incident* or outcome*)).tw.
77 (abnormalit* or toxicit* or complication* or consequence* or noxious or tolerabilit*).tw.
78 (ae or po or co).fs.
79 or/74–78
80 63 and 79
81 80 not 73

##### A.5.4. Economics

84 exp economics/(258163)
85 exp financial management/(17991)
86 exp financial support/(168377)
87 exp “financing organized”/(51967)
88 exp “business”/(26100)
89 or/85–88(249186)
90 84 not 89(24912)
91 health resource allocation/(3423)
92 health resource utilization/(4982)
93 exp “Quality of Life”/(23733)
94 Patient Satisfaction/(14059)
95 (econom* or cost or costs or costly or costing or price or prices or pricing or budget*).ti,ab.(53804)
96 (expenditure* not energy).ti,ab.(2243)
97 (value adj2 money).ti,ab.(187)
98 (QOL or QOLY or QOLYs or HRQOL or QALY or QALYs).ti,ab.(3012)
99 or/90–98(107583)
100 63 and 99(255)
101 100 not (73 or 81)

#### A.6. MANTIS <1880 to October 2008>

1 neck.de.
2 (spine or Cervical Vertebrae or Coccyx or Intervertebral Disk or Lumbar Vertebrae or Sacrum or Spinal Canal or Thoracic Vertebrae).de.
3 (Back or Lumbosacral Region or Sacrococcygeal Region).de.
4 neck muscles.de.
5 Zygapophyseal Joint.de.
6 or/1–5
7 pain.de.
8 pain, intractable.de.
9 pain, referred.de.
10 (pain* or ache* or aching).tw.
11 or/7–10
12 6 and 11
13 (back pain or low-back pain).de.
14 back injuries.de.
15 (backpain* or backache*).tw.
16 (spinal injuries or spinal fractures).de.
17 (spinal diseases or Intervertebral Disk Displacement or Spinal Stenosis or Spondylolisthesis or Spondylolysis).de.
18 ((disc* or disk*) adj3 (degener* or displace* or prolapse* or hernia∗ or bulge or protrusion* or extrusion* or sequestration* or disorder* or disease* or rupture* or slipped)).tw.
19 ((stenosis or stenoses) adj3 (lumbar or spine or spines or spinal)).tw.
20 (Spondylolys* or spondylolisthes* or Spondylisthes*).tw.
21 (Discitis or diskitis or Spondylodis*).tw.
22 (osteoporo* adj3 compression fracture*).tw.
23 vertebrogenic pain syndrome*.tw.
24 Sciatica.de.
25 (Sciatica or ischialgia).tw.
26 (Sciatic adj3 (Neuralgia or Bilateral)).tw.
27 neck pain.de.
28 (cervicalgia or Cervicodynia).tw.
29 ((anterior or posterior) adj3 (cervical pain or cervical ache*)).tw.
30 ((cervicogenic or cervicogenic) adj3 headache*).tw.
31 (neck injuries or Whiplash Injuries).de.
32 (neckache* or neckpain*).tw.
33 (whiplash* or whip lash* or radiculomyelopath* or radiculo-myelopath*).tw.
34 (neck disorder* adj3 radicul*).tw.
35 failed back surgery.de.
36 (failed back or back surgery syndrome* or FBSS).tw.
37 facet syndrome.de.
38 ((Zygapophyseal or Facet or facets) adj3 (syndrome* or degenerat*)).tw.
39 ((back or neck or spine or spinal or lumbar* or thoracic) adj3 (ache* or aching or pain* or strain*)).tw.
40 (lumbago or dorsalgia).tw.
41 (myofascial pain syndromes or myofascial).de.
42 (myofascial adj3 (pain* or ache*)).tw.
43 or/12–42
44 Acupuncture.de.
45 Acupuncture Therapy.de.
46 electroacupuncture.de.
47 (Acupuncture or acu-puncture or electroacupuncture or electro-acupuncture or electric acupuncture or electric acu-puncture or needling or acupressure or acu-pressure or mox?bustion).tw.
48 Manipulation, Spinal.de.
49 Manipulation, Chiropractic.de.
50 Chiropractic.de.
51 ((back or neck or spine or spinal or lumbar or cervical or chiropractic* or musculoskeletal* or musculo-skeletal*) adj3 (adjust* or manipulat* or mobiliz* or mobilis*)).tw.
52 (Manual adj therap*).tw.
53 (Manipulati* adj (therap* or medicine)).tw.
54 (Massage or Acupressure).de.
55 (massag* or reflexolog* or rolfing or zone therap*).tw.
56 (Chih Ya or Shiatsu or Shiatzu or Zhi Ya).tw.
57 (Flexion adj2 distraction*).tw.
58 (myofascial adj3 (release or therap*)).tw.
59 Muscle energy technique*.tw.
60 Trigger point*.tw.
61 Proprioceptive Neuromuscular Facilitation*.tw.
62 Cyriax Friction.tw.
63 (Lomilomi or lomi-lomi or trager).tw.
64 Aston patterning.tw.
65 (Strain adj counterstrain).tw.
66 Alexander technique*.tw.
67 (Craniosacral Therap* or Cranio-sacral Therap*).tw.
68 (amma or ammo or Effleurage or Petrissage or hacking or Tapotment).tw.
69 Complementary Therapies.de.
70 ((complement* or alternat* or osteopathic*) adj (therap* or medicine)).tw.
71 (Tui Na or Tuina).tw.
72 or/44–71
73 43 and 72

*The following filters were applied and overlap removed:*

##### A.6.1. Randomized/Controlled Clinical Trials

74 (Randomized Controlled Trial or Randomized Controlled Trials).de.
75 (Controlled Clinical Trial or Controlled Clinical Trials).de.
76 (random* or sham or placebo*).tw.
77 placebos.de.
78 Random Allocation.de.
79 Single Blind Method.de.
80 Double Blind Method.de.
81 ((singl* or doubl* or tripl* or trebl*) adj (blind* or dumm* or mask*)).tw.
82 (RCT or RCTs).tw.
83 (control* adj2 (study or studies or trial*)).tw.
84 or/74–83
85 animal.de.
86 human.de.
87 85 not (85 and 86)
88 73 and 84
89 88 not 87

##### A.6.2. Systematic Review

90 Meta-Analysis.de.
91 (meta analy* or metaanaly* or met analy* or metanaly*).tw.
92 (collaborative research or collaborative review* or collaborative overview*).tw.
93 (integrative research or integrative review* or integrative overview*).tw.
94 (quantitative adj3 (research or review* or overview*)).tw.
95 (research integration or research overview*).tw.
96 (systematic* adj3 (review* or overview*)).tw.
97 (methodologic* adj3 (review* or overview*)).tw.
98 Technology Assessment, Biomedical.de.
99 (hta or htas or technology assessment*).tw.
100 ((hand adj2 search*) or (manual* adj search*)).tw.
101 ((electronic adj database*) or (bibliographic* adj database*)).tw.
102 ((data adj2 abstract*) or (data adj2 extract*)).tw.
103 (Data adj3 (pool or pooled or pooling)).tw.
104 (Analys* adj3 (pool or pooled or pooling)).tw.
105 Mantel Haenszel.tw.
106 (Cochrane or PubMed or MEDLINE or EMBASE or PsycINFO or PsycLIT or PsychINFO or PsychLIT or CINAHL or Science Citation Index).ab.
107 or/90–106
108 73 and 107
109 108 not 87
110 109 not 89

##### A.6.3. Safety

90 (safe or safety or unsafe).tw.
91 (side effect* or side event*).tw.
92 ((adverse or undesirable or harm* or injurious or serious or toxic) adj3 (effect* or reaction* or event* or incident* or outcome*)).tw.
93 (abnormalit* or toxicit* or complication* or consequence* or noxious or tolerabilit*).tw.
94 adverse effects.de.
95 complications.de.
96 toxicity.de.
97 or/90–96
98 73 and 97
99 98 not 87
100 99 not 89

##### A.6.4. Economics

101 economics.de.
102 “costs and cost analysis”.de.
103 “value of life”.de.
104 economics, medical.de.
105 (econom* or cost or costs or costly or costing or price or prices or pricing).ti,ab.
106 (expenditure* not energy).ti,ab.
107 (value adj2 money).ti,ab.
108 budget.ti,ab.
109 cost benefit analysis.de.
110 or/101–109
111 73 and 110
112 111 not 87
113 112 not (89 or 100)

#### A.7. Cochrane Library 2009 Issue 2

##### A.7.1. Systematic Review and RCT/CCT

#1 MeSH descriptor Neck explode all trees
#2 MeSH descriptor Spine explode all trees
#3 MeSH descriptor Back explode all trees
#4 MeSH descriptor Neck Muscles explode all trees
#5 MeSH descriptor Zygapophyseal Joint explode all trees
#6 MeSH descriptor Pain explode all trees
#7 MeSH descriptor Pain, Intractable explode all trees
#8 MeSH descriptor Pain, Referred explode all trees
#9 (pain* or ache*):ti,ab,kw
#10 #1 OR #2 OR #3 OR #4 OR #5
#11 #6 OR #7 OR #8 OR #9
#12 #10 AND #11
#13 MeSH descriptor Back Pain explode all trees
#14 MeSH descriptor Back Injuries explode all trees
#15 (backpain* or backache*):ti,ab,kw
#16 MeSH descriptor Spinal Injuries explode all trees
#17 MeSH descriptor Spinal Diseases explode all trees
#18 (disc* or disk*) NEAR/3 (degener* or displace* or prolapse* or hernia* or bulge or protrusion* or extrusion* or sequestration* or disorder* or disease* or rupture* or slipped):ti,ab,kw
#19 (stenosis or stenoses) NEAR/3 (lumbar or spine or spines or spinal):ti,ab,kw
#20 (Spondylolys* or spondylolisthes* or Spondylisthes*):ti,ab,kw or (Discitis or diskitis or Spondylodis*):ti,ab,kw
#21 (osteoporo* NEAR/3 compression fracture*):ti,ab,kw
#22 (vertebrogenic pain syndrome*):ti,ab,kw
#23 MeSH descriptor Sciatica explode all trees
#24 (Sciatica or ischialgia):ti,ab,kw or (Sciatic NEAR/3 (Neuralgia or Bilateral)):ti,ab,kw
#25 MeSH descriptor Neck Pain explode all trees
#26 (cervicalgia or Cervicodynia):ti,ab,kw or (anterior or posterior) NEAR/3 (cervical pain or cervical ache*):ti,ab,kw or (cervicogenic or cervicogenic) NEAR/3 headache*:ti,ab,kw
#27 MeSH descriptor Neck Injuries explode all trees
#28 (neckache* or neckpain*):ti,ab,kw or (whiplash* or whip lash* or radiculomyelopath* or radiculo-myelopath*):ti,ab,kw or (failed back or back surgery syndrome* OR FBSS):ti,ab,kw or (lumbago or dorsalgia):ti,ab,kw
#29 (neck disorder*) NEAR/3 radicul*:ti,ab,kw or (Zygapophyseal or Facet or facets) NEAR/3 (syndrome* or degenerat*):ti,ab,kw or (back or neck or spine or spinal or lumbar* or thoracic) NEAR/3 (ache* or aching or pain* or strain*):ti,ab,kw or (myofascial adj3 (pain* or ache*)):ti,ab,kw
#30 #12 OR #13 OR #14 OR #15 OR #16 OR #17 OR #18 OR #19 OR #20 OR #21 OR #22 OR #23 OR #24 OR #25 OR #26 OR #27 OR #28 OR #29
#31 MeSH descriptor Acupuncture explode all trees
#32 MeSH descriptor Acupuncture Therapy explode all trees
#33 MeSH descriptor Electroacupuncture explode all trees
#34 (acupuncture or electric acupuncture or electric acu-puncture or needling or acupressure or acu-pressure or mox?bustion):ti,ab,kw
#35 MeSH descriptor Manipulation, Spinal explode all trees
#36 MeSH descriptor Manipulation, Chiropractic explode all trees
#37 MeSH descriptor Chiropractic explode all trees
#38 (back or neck or spine or spinal or lumbar or cervical or chiropractic* or musculoskeletal* or musculo-skeletal*) NEAR/3 (adjust* or manipulat* or mobiliz* or mobilis*):ti,ab,kw or (Manual NEXT therap*):ti,ab,kw or (Manipulati* NEXT (therap* or medicine)):ti,ab,kw
#39 MeSH descriptor Massage explode all trees
#40 (massag* or reflexolog* or rolfing or zone therap*):ti,ab,kw or (Chih Ya or Shiatsu or Shiatzu or Zhi Ya):ti,ab,kw or (Flexion NEAR/2 distraction*):ti,ab,kw or (myofascial NEAR/3 (release or therap*)):ti,ab,kw or (Muscle energy technique*):ti,ab,kw
#41 (Trigger point*):ti,ab,kw or (Proprioceptive Neuromuscular Facilitation*):ti,ab,kw or (Cyriax Friction):ti,ab,kw or (Lomilomi or lomi-lomi or trager or Tui Na or Tuina):ti,ab,kw or (Aston patterning):ti,ab,kw
#42 (Strain NEAR/1 counterstrain):ti,ab,kw or (Alexander technique*):ti,ab,kw or (Craniosacral Therap* or Cranio-sacral Therap*):ti,ab,kw or (amma or ammo or Effleurage or Petrissage or hacking or Tapotment):ti,ab,kw or (complement* or alternat* or osteopathic*) NEXT (therap* or medicine):ti,ab,kw
#43 MeSH descriptor Complementary Therapies, this term only
#44 #31 OR #32 OR #33 OR #34 OR #35 OR #36 OR #37 OR #38 OR #39 OR #40 OR #41 OR #42 OR #43
#45 #30 AND #44

##### A.7.2. Safety

#46 Any MeSH descriptor with qualifier: AE
#47 Any MeSH descriptor with qualifier: TO
#48 Any MeSH descriptor with qualifier: PO
#49 Any MeSH descriptor with qualifier: CO
#50 (safe or safety or unsafe):ti,ab,kw or (side effect* or side event*):ti,ab,kw or (adverse or undesirable or harm* or injurious or serious or toxic) NEAR/3 (effect* or reaction* or event* or incident* or outcome*):ti,ab,kw or (abnormalit* or toxicit* or complication* or consequence* or noxious or tolerabilit*):ti,ab,kw
#51 #46 OR #47 OR #48 OR #49 OR #50
#52 #45 AND #51

##### A.7.3. Economics

#53 MeSH descriptor Economics, this term only
#54 MeSH descriptor Economics, Medical, this term only
#55 MeSH descriptor Costs and Cost Analysis explode all trees
#56 MeSH descriptor Value of Life explode all trees
#57 MeSH descriptor Quality-Adjusted Life Years explode all trees
#58 MeSH descriptor Patient Satisfaction explode all trees
#59 Any MeSH descriptor with qualifier: EC
#60 (econom* or cost or costs or costly or costing or price or prices or pricing or budget*):ti,ab,kw or (expenditure* not energy):ti,ab,kw or (value NEAR/2 money):ti,ab,kw or (QOL or QOLY or QOLYs or HRQOL or QALY or QALYs):ti,ab,kw
#61 #53 OR #54 OR #55 OR #56 OR #57 OR #58 OR #59 OR #60
#62 #45 AND #61
#63 #62 AND NOT #52

#### A.8. Index to Chiropractic Literature 2008 Oct 10

S1 Subject: “BACK PAIN” OR “BACK INJURIES” OR “NECK INJURIES” OR “NECK PAIN” OR “SPINAL DISEASES” OR “SPINAL INJURIES” OR “SCIATICA” OR All Fields:backpain* or backache* OR “back pain" OR “back ache” OR “back pains” OR “back aches” OR neckpain* OR neckache* OR “neck pain” OR neck ache“ OR ”neck pains“ OR ”neck aches“ OR All Fields:Spondylolys* or spondylolisthes* or Spondylisthes* or Discitis or diskitis or Spondylod* OR Sciatica OR ischialgia* OR cervicalgia OR Cervicodynia
S2 All Fields: whiplash* or “whip lash" OR “whip lashes" or radiculomyelopath* or “radiculo-myelopathy” OR “radiculo-myelopathies” OR All Fields: “failed back” or “back surgery syndrome” or “back surgery syndromes” or FBSS OR All Fields:lumbago or dorsalgia or “myofascial pain” OR “myofascial ache”
S3 All Fields: “cervical pain” OR “cervical ache” OR “vertebrogenic pain syndrome” OR “vertebrogenic pain syndromes” OR All Fields: “degenerated disk” OR “degenerative disk” OR “degenerated disks” OR “degenerative disks” OR All Fields: “degenerated disc” OR “degenerative disc” OR “degenerated discs” OR “degenerative discs”
S4 All Fields: “prolapsed disk” OR “prolapsed disks” OR “prolapsed disc” OR “prolapsed discs” OR “disk prolapse” OR “disc prolapse” “herniated disk” OR “herniated disks” OR “herniated disc” OR “herniated discs” OR All Fields: “displaced disk” OR “displaced disks” OR “displaced disc” OR “displaced discs” OR “osteoporotic compression fracture” OR “osteoporotic compression fractures” OR All Fields:: “lumbar stenosis” OR “lumbar stenoses” OR “spinal stenosis” OR “spinal stenoses” OR “cervicogenic headache” OR “cervicogenic headaches” OR “cervicogenic headache” OR “cervicogenic headaches”
S5 All Fields: radiculomyelopathy OR radiculomyelopathies OR “radiculo-myelopathy” OR “radiculo-myelopathies” OR All Fields: “Zygapophyseal joint syndrome” OR “Zygapophyseal joint syndromes” OR “Z-joint syndrome” OR “Z-joint syndromes” OR “facet joint syndrome” OR “facet joint syndromes” OR All Fields: “thoracic pain” OR “thoracic ache” OR “spinal pain” OR “spinal ache” OR “lumbar pain” OR “lumbar ache”
S6 S1 OR S2 OR S3 OR S4 OR S5
S7 Subject: “ACUPUNCTURE” OR “ACUPRESSURE” OR “ACUPUNCTURE THERAPY” OR “ELECTROACUPUNCTURE” OR “MANIPULATION, LUMBAR” OR “MANIPULATION, CERVICAL” OR “MANIPULATION, CHIROPRACTIC” OR “MANIPULATION, SPINAL” OR “MANIPULATION, THORACIC” OR Subject: “MASSAGE” OR “CHIROPRACTIC” OR All Fields:acupuncture or “acu-puncture” or electroacupuncture or “electro-acupuncture” or “electric acupuncture” or “electric acu-puncture” or needling or acupressure or “acu-pressure” or moxibustion or moxabustion
S8 All Fields: “manual therapy” OR “manual therapies” OR massag* or reflexolog* or rolfing or “zone therapy” or “zone therapies” OR All Fields: “Chih Ya” or Shiatsu or Shiatzu or “Zhi Ya” or “Flexion distraction” OR “Trigger point” OR “Trigger points” OR “Proprioceptive Neuromuscular Facilitation” OR “Proprioceptive Neuromuscular Facilitations” OR All Fields: “myofascial release” or “myofascial therapy” OR “myofascial therapies” OR “Muscle energy technique” OR “Muscle energy techniques” OR “Cyriax Friction”
S9 All Fields: Lomilomi or “lomi-lomi” or trager or “Aston patterning” or “Strain counterstrain” or “Alexander technique” or “Alexander techniques” or “Tui Na” or Tuina OR All Fields: “Craniosacral Therapy” or “Craniosacral Therapies” or “Cranio-sacral Therapy” or “Cranio-sacral Therapies” or amma or ammo or Effleurage or Petrissage or hacking or Tapotment OR All Fields:manipulat* or mobiliz* or mobilis*
S10 All Fields: “complementary therapy” OR “complementary therapies” OR “complementary medicine” OR All Fields: “alternative therapy” OR “alternative therapies” OR “alternative medicine” OR All Fields: “osteopathic therapy” OR “osteopathic therapies” OR “osteopathic medicine”
S11 S7 OR S8 OR S9 OR S10
S12 S6 AND S11

##### A.8.1. Randomized/Controlled Clinical Trials

S13 Publication Type: Randomized Controlled Trial
S14 Subject: “RANDOMIZED CONTROLLED TRIALS AS TOPIC” OR “CONTROLLED CLINICAL TRIALS” OR “PLACEBOS” OR All Fields:random* or sham or placebo* or RCT or RCTs or CCT or CCTs OR All Fields: “controlled clinical trial” or “controlled clinical trials” or “controlled study” or “controlled studies” or “control study” or “controlled studies”
S15 S12 AND S14
S16 S13 OR S15

##### A.8.2. Safety

S17 All Fields:safe or safety or unsafe or “side effect” or “side effects” or “side event” or “side events” OR All Fields:abnormalit* or toxicit* or complication* or consequence* or noxious or tolerabilit* OR All Fields:adverse or undesirable or harm* or injurious or serious or toxic
S18 S12 AND S17

##### A.8.3. Economics

S19 Subject: “ECONOMICS” OR “ECONOMICS, MEDICAL” OR “COSTS AND COST ANALYSIS” OR All Fields:econom* or cost or costs or costly or costing or price or prices or pricing or budget* or expenditure or value or money
S20 S12 AND S19

#### A.9. LILACS 2008 Oct 13

(((((“BACK PAIN” or “NECK PAIN”) or “SPINAL DISEASES”) or “BACK INJURIES”) or “SPINAL INJURIES”) or “NECK INJURIES”) or “SCIATICA” [Descritor de assunto] and acupuncture or electroacupuncture or acupressure or massage or manipulation or chiropractic or osteopathic [Palavras]

#### A.10. Acubriefs 2008 Oct 10

KW: Back pain + SPECIALTY: RCT/randomized controlled trials
KW: neck pain + SPECIALTY: RCT/randomized controlled trials
KW: thoracic pain + SPECIALTY: RCT/randomized controlled trials
KW: spinal diseases + SPECIALTY: RCT/randomized controlled trials
KW: lumbago + SPECIALTY: RCT/randomized controlled trials
KW: facet joint + SPECIALTY: RCT/randomized controlled trials

*
Excluded PubMed refs, ACP Jnl Club, Cochrane, ClinicalTrials.gov, animal studies*

### B. Evidence Tables

See Tables 2–11.
